# Effects of Glutathione-Enriched Inactive Dry Yeast on the Flavor Profile of Kiwi Wine

**DOI:** 10.3390/foods14101760

**Published:** 2025-05-15

**Authors:** Zhibo Yang, Chuan Song, Qiuyu Lan, Bin Hu, Zonghua Ao, Suyi Zhang, Junni Tang, Xin Du, Chenglin Zhu, Luca Laghi

**Affiliations:** 1 College of Pharmacy and Food, Southwest Minzu University, Chengdu 610041, China; yangzhibo@stu.sicau.edu.cn (Z.Y.); junneytang@swun.edu.cn (J.T.); 2 College of Food Science, Sichuan Agricultural University, Ya’an 625014, China; hubin2555@sina.com; 3 Luzhou Laojiao Co., Ltd., Luzhou 646000, China; songchuan@lzlj.com (C.S.); aozh@lzlj.com (Z.A.); zhangsy@lzlj.com (S.Z.); 4 Department of Agricultural and Food Sciences, University of Bologna, 47521 Cesena, Italy; lanqiuyu211@gmail.com (Q.L.); l.laghi@unibo.it (L.L.); 5 College of Chemistry and Life Sciences, Chengdu Normal University, Chengdu 611130, China; 081056@ednu.edu.cn

**Keywords:** kiwi wine, glutathione-enriched inactive dry yeast, aroma compounds, taste compounds, multivariate analysis

## Abstract

This study aimed to explore the influence of glutathione-enriched inactive dry yeast (g-IDY) addition on the changes in aroma and taste compounds in kiwi wine (KW), produced from green-, red-, and yellow-flesh kiwifruits. In total, 42 aroma compounds and 67 taste compounds were characterized using GC-IMS and ^1^H-NMR, respectively. Among them, six aroma compounds and thirty-one taste compounds were determined as key compounds based on a *t*-test, variable importance in projection scores, and relative odor activity value. Results indicated that g-IDY addition significantly decreased the concentration of hexyl acetate and increased the concentrations of 1-hexanol-M and pentanal in KW produced from green- and yellow-flesh kiwifruits. Pearson correlation analysis revealed strong associations between key aroma and taste compounds, particularly highlighting significant negative correlations between amino acids and aroma compounds. The findings could shed light on KW processing optimization and provide theoretical support for integrating g-IDY into KW industrial production.

## 1. Introduction

Glutathione (GSH), a tripeptide composed of L-glutamate, L-cysteine, and glycine, offers a protective effect on desirable wine aromas, mitigates the formation of off-flavors, and reduces the formation of browning pigments [1,2]. Considering its beneficial impact on wine quality, the International Organization of Vine and Wine (OIV) has endorsed the treatment of must and wine with GSH [3]. However, the application of GSH in winemaking is constrained by regulatory dosage limits and high production costs. Consequently, there is a pressing necessity to explore and implement viable alternatives to pure GSH in the wine industry.

Inactive dry yeast (IDY) refers to yeast that has undergone thermal inactivation after cultivation under aerobic conditions in sugar-rich media. According to the manufacturing processes, commercially available IDY products can be categorized into four types: inactive yeast, yeast autolysates, yeast hulls or walls, and yeast extracts [4]. Among these products, glutathione-enriched IDY (g-IDY) is commonly utilized in brewing as a substitute for pure GSH. Research has shown that g-IDY can either release GSH directly into wine and/or provide precursors for GSH synthesis, thereby increasing the GSH content in the wine [5]. Additionally, g-IDY has been observed to release complex nitrogen-containing volatile compounds into wine, influencing its aroma profile [6].

Kiwi wine (KW), a fermented alcoholic beverage produced from kiwifruit through yeast-driven alcoholic fermentation, is nicely tailored for consumers seeking low-alcohol beverages, given its inherently low alcohol content, suggesting substantial market potential for growth [7]. The differences in kiwifruit material come from their variety. Various varieties may exhibit different flesh colors, which can be green, red, or yellow, and there are significant differences in the flavor of KW produced by different kiwifruit varieties [8]. The flavor profile of KW encompasses both its aroma and taste profiles, which are crucial factors influencing consumer preferences [9]. Aroma, primarily shaped by volatile compounds, such as alcohols, aldehydes, esters, acids, and phenols, plays a pivotal role in defining the organoleptic attributes of KW [10]. Additionally, taste compounds, including organic acids and free amino acids, also significantly contribute to the aroma and taste profiles of wines through various chemical, biochemical, and sensory interactions [4]. For instance, during wine fermentation, amino acids could enhance the complexity of flavor by imparting freshness, sweetness, bitterness, and astringency, directly or through conversion into volatile compounds [11].

The relationship between amino acids and volatile components in KW fermented by green-flesh kiwifruit with the addition of g-IDY was investigated in a previous study [4]. Meanwhile, in terms of taste compounds, apart from amino acids, KW contains a large number of organic acids, sugars, and polyphenolic compounds, and the effect of the addition of g-IDY on these substances is unknown. In order to fill such gaps, this study aimed to investigate the effect of g-IDY addition on the aroma and taste profiles dominated by volatile and non-volatile compounds in KW produced from green-, yellow-, and red-flesh kiwifruits. The raw materials were selected from the most representative and currently common kiwifruit varieties with different flesh colors, namely Hayward (green flesh), Donghong (red flesh), and Jinshi (yellow flesh). Several analytical techniques, previously singularly employed, were here combined to capture the comprehensive sensory characteristics and flavor profiles of KW, namely gas chromatography–ion mobility spectrometry (GC-IMS), proton nuclear magnetic resonance spectroscopy (^1^H-NMR), and electronic tongue analysis [12,13]. Meanwhile, multivariate analyses, such as principal component analysis (PCA) and partial least squares discriminant analysis (PLS-DA), were combined to discern variations in the aroma, sensory attributes, and taste properties of KW [14]. The findings could shed light on KW processing optimization and provide theoretical support for integrating g-IDY in KW industrial production.

## 2. Materials and Methods

### 2.1. Materials

Fruits from three cultivars, namely Hayward (green flesh), Donghong (red flesh), and Jinshi (yellow flesh), fully ripe and each weighing approximately 100 g, were collected for this study. The initial sugar content of kiwifruit was 10 °Brix, and the juice yield was 75%. These kiwifruits were harvested in September 2023 from a plantation in Pujiang, Sichuan, China, as shown in Figure S1. Following a previous study of ours [15], the yeast strain employed was *S. cerevisiae* strain RW (Angel Yeast Co., Ltd., Yichang, Hubei, China), selected for its well-documented fermentation characteristics for KW fermentation.

### 2.2. KW Samples

**Figure 1:**
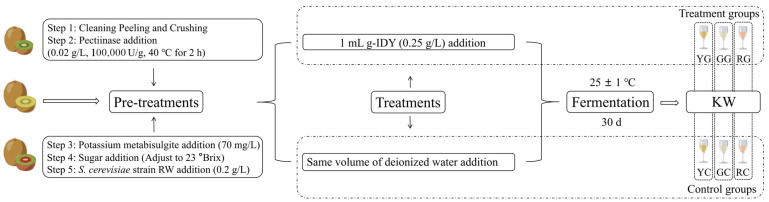
KW production workflow.

All kiwifruits collected for this study were transported to the laboratory right after harvesting. KW production procedures were conducted on a laboratory scale, as illustrated in Figure 1. In the pretreatment phase, the kiwifruits underwent cleaning, peeling, and crushing. Enzymatic digestion was then performed using pectinase (0.02 g/L, 100,000 U/g, SAS SOFRALAB, Magenta, France) for 2 h. The enzyme digestion temperature was controlled at 40 °C by an HH-8 thermostatic water bath (GUOHUA INSTRUMENT MANUFACTURING Co., Ltd., Changzhou, China). Subsequently, potassium metabisulfite (70 mg/L, Shanghai Yuanye Bio-Technology Co., Ltd., Shanghai, China) was added to prevent browning, and sucrose was incorporated to adjust the initial sugar content to 23 °Brix. Finally, activated yeast (0.2 g/L, Saccharomyces cerevisiae RW, Angel Yeast Co., Ltd., Yichang, Hubei, China) was introduced. Prior to fermentation, 1 mL of g-IDY (0.25 g/L) was added to each treated sample, while an equal volume of deionized water was added to samples used as controls. The total fermentation volume was 200 mL, and fermentation was conducted at 25 ± 1 °C for 30 days through a DHP-9162D thermostat (Keelrein Instrument Co., Ltd., Shanghai, China). Each group underwent five replicates. After fermentation, the KW samples were filtered through three layers of sterile gauze and stored at −80 °C until further analysis.

### 2.3. GC-IMS Analysis

Following Zhang et al. [15], headspace analysis was conducted using a GC-IMS instrument (Flavourspe^®^, G.A.S. Dortmund Company, Dortmund, Germany), equipped with a syringe and an auto-sampler unit. A Restek MXT-5 column (30 m × 0.53 mm × 1 μm) was employed in GC, with the column and IMS temperatures set at 60 °C and 45 °C, respectively. Samples (1.5 mL) were transferred into a 20 mL headspace glass sampling vial and incubated at 60 °C for 10 min. Subsequently, 200 µL of headspace samples were injected into the injector, operating at 85 °C in splitless mode using a heated syringe. Nitrogen gas (99.999% purity) served as carrier/drift gas, with EPC1 (IMS drift gas) maintained at a flow rate of 150 mL/min. The flow rates were programmed as follows: 2 mL/min for 5 min, 10 mL/min for 10 min, 15 mL/min for 5 min, 50 mL/min for 10 min, and 100 mL/min for 10 min. The qualitative analysis of volatile aroma compounds relied on IMS, supported by the NIST database, integrated within the GC-IMS Library Search. Quantitative analysis was based on peak intensities by means of rectangular integration. The Laboratory Analysis Viewer and Reporter provided by the GC-IMS instrument was utilized to generate fingerprints of each sample.

### 2.4. Relative Odor Activity Value

The relative odor activity value (ROAV) of each flavor compound was calculated according to the methodology described in previous studies [16] and is detailed in the Supporting Material.

### 2.5. ^1^H-NMR Analysis

The ^1^H-NMR analysis was performed through a previously established protocol [17]. Solid residues were removed by centrifuging 0.5 mL of each sample at 18,630× *g* and 4 °C for 15 min. The resulting supernatant (0.35 mL) was then mixed with bi-distilled water (0.35 mL) and a 200 μL NMR analysis solution (D2O solution of 3-(trimethylsilyl)-propionic-2,2,3,3-d4 acid sodium salt (10 mmol/L) + phosphate buffer (1 mol/L) + NaN_3_ (2 mmol/L)), followed by a centrifugation step.

^1^H-NMR spectra of KW samples were recorded using a 600.13 MHz AVANCE III spectrometer (Bruker, Wuhan, China) at 298 K, controlled by Topspin 4.2 software. Experimental conditions are detailed in Figure S2. Following Yang et al. [17], spectral phase adjustment and data processing were performed using the R language. Baseline correction utilized the “rolling ball” algorithm from the R (v.4.4.2) baseline package, and probabilistic quotient normalization (PQN) was applied to normalize molecule concentrations. Compound identification relied on the comparison of peak multiplicity and chemical shifts with standard compound spectra from the Chenomx library (Chenomx Inc., Edmonton, AB, Canada, v.10.1). Signal area was calculated using rectangular integration.

### 2.6. E-Tongue

As detailed in a previous study of ours [18], the analysis of all samples was performed using an α-Astree E-tongue (Alpha MOS, Toulouse, France). The sensors employed were specifically sensitive to sweetness (ANS), saltiness (CTS), umami (NMS), sourness (AHS), and bitterness (SCS).

In total, 80 mL of a KW sample was transferred into a designated beaker for each E-tongue analysis. Data acquisition time, stirring rate, and analysis duration were set at 120 s, 60 rpm/min, and 3 min, respectively. After each test, the sensors were thoroughly rinsed with deionized water for 30 s. Output values were recorded between 100 and 120 s. Eight test replicates for each sample were conducted, and the mean value of the final five stable measurements was selected for subsequent data analysis.

### 2.7. Statistical Analysis

The R language was utilized to perform a *t*-test (*p* < 0.05). Data distribution was normalized using the Box and Cox method [19] prior to conducting univariate analyses. Principal component analysis (PCA) and projections to latent structures–discriminant analysis (PLS-DA) were conducted using MetaboAnalyst 6.0 https://www.metaboanalyst.ca (accessed on 20 March 2025). Additionally, the OmicStudio tools https://www.omicstudio.cn/tool (accessed on 20 March 2025) were employed for Mantel tests and the construction of correlation networks.

## 3. Results and Discussion

### 3.1. Key Aroma Compounds in KW

**Figure 2:**
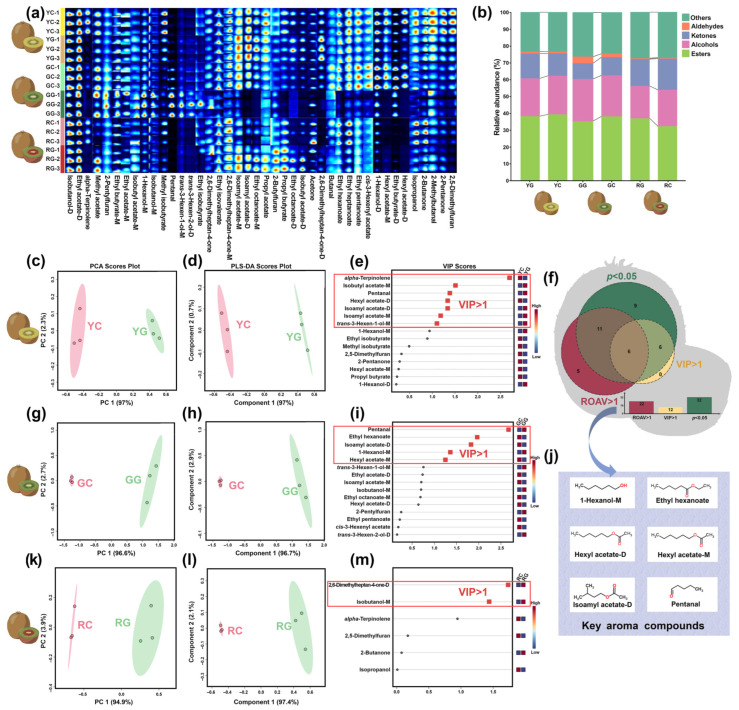
Fingerprint plot (**a**) and bar chart (**b**) representing the variation in the concentration of molecules in different groups. PCA scores plot (**c**,**g**,**k**), PLS-DA scores plot (**d**,**h**,**l**), and VIP scores (**e**,**i**,**m**) of the aroma compounds in the KW samples. Venn plot of *p* < 0.05, ROAV > 1, and VIP > 1 of the aroma compounds in KW produced with/without the addition of g-IDY (**f**). The key aroma compounds for KW (**j**).

Information regarding the identification and analysis of aroma components through GC-IMS is depicted in Figure 2 and Table 1.

As shown in Figure 2a, a total of 42 aroma compounds were characterized by GC-IMS, comprising esters (22), alcohols (7), aldehydes (3), ketones (6), and others (4), as detailed in Table S1. PCA models were developed for each kiwifruit variety in order to assess the influence of the g-IDY addition on the aroma compounds of KW, as shown in Figure 2c,g,k. The first principal component (PC 1) explained 97%, 94.6%, and 94.9% of the overall variance, respectively, thus nicely summarizing the flavor differences between the KW produced with/without g-IDY. PLS-DA models demonstrated strong predictive capability, as can be visually appreciated in Figure 2d,h,l. The VIP scores from PLS-DA were used to rank the contribution of variables to classification (Figure 2e,i,m). As depicted in Figure 2f, 12 aroma compounds, whose VIP scores are greater than 1, were identified as contributing significantly to classification, as suggested by Wang et al. [20]. The perception of aroma compounds in KW is influenced by both their concentration and threshold. Relative odor activity value (ROAV) analysis was used to identify key aroma compounds by considering their threshold. Ethyl acetate, recognized for its fruity–sweet aroma [21], was selected as a reference due to its high relative content (8.7%) and low threshold (0.005 mg/kg), underscoring its significant impact on KW’s overall aroma profile. Compounds with ROAV > 1 are major contributors to flavor, while those with a ROAV between 0.1 and 1 can modify the overall aroma. Table S1 lists 22 aroma compounds with ROAVs exceeding 1, predominantly esters, emphasizing their pivotal roles in shaping KW’s aroma characteristics. Figure 2j illustrates six key aroma compounds filtered based on the criteria of ROAV > 1, *p* < 0.05, and VIP > 1, namely 1-hexanol-M, ethyl hexanoate, hexyl acetate-D, hexyl acetate-M, isoamyl acetate-D, and pentanal.

Esters are mainly generated through substrate esterification and yeast enzymatic metabolism during fermentation [22]. Four esters were identified as key aroma compounds in KW, including ethyl hexanoate, hexyl acetate-D, hexyl acetate-M, and isoamyl acetate-D. Ethyl hexanoate, partly derived from kiwifruit itself [23], exhibited high ROAV in all samples, ranging from 9.70 to 49.45, contributing floral and fruity notes to KW, in agreement with a previous study [24]. Notably, a significantly higher level was only found in KW produced from Hayward (green flesh) kiwifruit with added g-IDY. Previous studies have demonstrated that KW produced by Hayward kiwifruits have the most pronounced aroma among KW produced with different flesh color kiwifruits, which may explain the phenomenon described above [8,25]. Hexyl acetate is formed through the reaction of ethanol with acetyl CoA, amino acids, carbohydrates, and lipids [26]. Its concentration was significantly lower in KW produced from green- and yellow-flesh kiwifruits with added g-IDY, compared to their control counterparts. Such a phenomenon could be a consequence of the content of ethanol in KW, the immediate precursor substance of hexyl acetate through esterification, whose trends were indeed identical in the above groups. On the other hand, the variation in its levels may also be linked to the presence of GSH derived from the g-IDY addition on the distinctive activities of some enzymes (such as ethanol dehydrogenase), which are involved in esters’ synthesis and breakdown. A previous study has confirmed that the g-IDY addition could prevent acetaldehyde from being oxidized to the corresponding carboxylic acid, indirectly inhibiting the esterification of alcohols and acids [3]. Isoamyl acetate, imparting desirable banana and pear aromas to KW, was identified as a key aroma compound of KW in previous research [27]. Its levels were significantly decreased in KW produced by green-flesh kiwifruits with added g-IDY, while the opposite was found in KW from yellow kiwifruits with added g-IDY.

Alcohols, contributing to the harmony of the KW aroma, are synthesized through glycolytic pathways, methyl ketone reduction, and amino acid metabolism [28]. In this study, significantly increased levels of 1-hexanol were found in KW produced from green- and yellow-flesh kiwifruits with added g-IDY, compared to their control counterparts. 1-hexanol, a higher alcohol, could be generated in yeast through glycolysis and the Ehrlich pathway [22]. Higher levels of GSH could be beneficial for higher alcohol formation by yeast metabolism, through the alteration of the redox state of cells. In detail, on one hand, GSH brought on by the g-IDY addition could promote the catabolism of the precursor amino acids, such as leucine [29], to produce higher alcohols. On the other hand, several substances (such as yeast cytosol and bicarb) released by g-IDY, could be used as nitrogen sources to promote the production of amino acids in yeast, and in turn to facilitate the formation of higher alcohols [30,31].

Aldehydes are typically formed by the oxidation of alcohols, serving as essential flavor substances in wines and harmonizing the aroma of the wine, even at low concentrations. The content of pentanal was significantly increased in KW produced from green- and yellow-flesh kiwifruits enriched with g-IDY, compared to their control groups. This could be linked to the oxidation of GSH brought on by the g-IDY addition, which promotes the release of free acetaldehyde [32]. 

### 3.2. Key Taste Components in KW

A total of 67 taste compounds were characterized by ^1^H-NMR, including alcohols and polyols (5), amino acids, peptides, and analogs (19), carbohydrates and carbohydrate conjugates (5), organic acids and derivatives (22), and others (15), as detailed in Table S2. A typical ^1^H-NMR spectrum of KW is shown in Figure S3.

**Figure 3:**
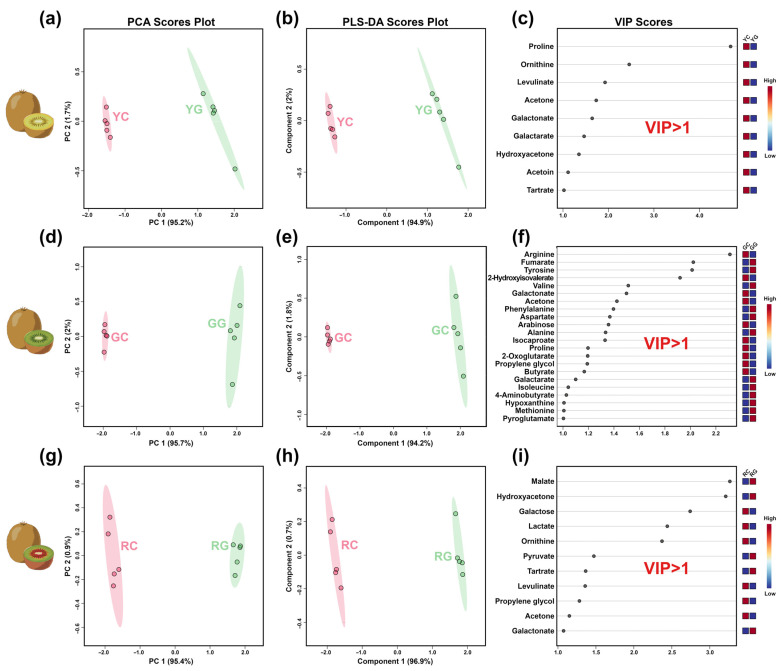
PCA score plots (**a**,**d**,**g**), PLS-DA score plots (**b**,**e**,**h**), and VIP scores (**c**,**f**,**i**) of the taste.

Similarly to aroma compounds, PCA models were calculated to assess the influence of the g-IDY addition on the taste compounds of KW, as shown in Figure 3a,d,g. For the three varieties of kiwifruit, the first principal component (PC 1) explained 95.2%, 95.7%, and 95.4% of the overall variance, respectively, thus nicely summarizing the taste differences between the KW produced with/without g-IDY. A total of 31 key taste compounds (*p* < 0.05 and VIP > 1) were identified, as illustrated in Figure 3c,f,i and Table 2.

Amino acids contribute considerably to the taste profile of KW and serve as precursors for some aroma compounds. Among the key taste compounds, eleven amino acids were identified, underscoring their critical role in determining KW’s taste profile. The concentrations of aspartate, isoleucine, phenylalanine, and valine were significantly higher in KW produced from green- and yellow-flesh kiwifruits with added g-IDY, compared to their controls. A parallel, opposite trend was found for proline and arginine. Both isoleucine and valine are crucial precursors for several aroma compound (such as isoamyl acetate and isobutyl acetate) formations by yeast during alcoholic fermentation via the Ehrlich pathway [33]. In our study, trends similar to those of isoamyl acetate and isobutyl acetate were found for valine and isoleucine in KW with added g-IDY, reinforcing the idea that the addition of g-IDY could impact the Ehrlich pathway in yeast.

Organic acids play pivotal roles in the taste profile of KW, imparting pleasant and refreshing sensorial attributes to it [34]. Among the key taste organic acids and derivatives identified in this study, the levels of fumarate, galactarate, and lactate were significantly increased in KW produced from green- and yellow-flesh kiwifruits with added g-IDY, while the levels of malate, 2-hydroxyisovalerate, 2-oxoglutarate, butyrate, and galactonate were significantly decreased. Previous studies have confirmed that lactate, produced by the conversion of malate during malolactic fermentation (MLF), could positively affect the taste profile of wine by adding complexity and balance to the mouthfeel [35]. In our study, the opposite trends observed for lactate and malate in KW were likely due to the beneficial effects of GSH produced by g-IDY on malate conversion during MLF, as reported by Torrea et al. [36].

### 3.3. E-Tongue Analysis

**Figure 4:**
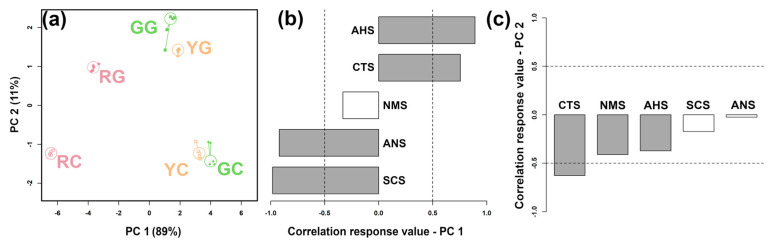
An rPCA model set up based on the response values of the E-tongue sensors representing the Scoreplot (**a**) and Loading plot (**b**,**c**). Gray bars (**b**) display significant correlations between the concentration of each molecule and its importance over the PC.

The E-tongue simulates human taste perception by detecting taste characteristics through electronic sensors, enabling an objective assessment of the taste profiles of samples [37]. As illustrated in Figure 4, the main driver of the distribution of KW samples along PC 1 is the varietal differences in kiwifruit, with KW produced by green- and yellow-flesh kiwifruits exhibiting positive PC 1 scores and KW produced by red-flesh kiwifruits exhibiting negative PC 1 scores. PC 2 mainly catches the differences associated with the addition of g-IDY, with KW produced with the addition of g-IDY appearing at positive PC 2 scores and KW without the addition appearing at negative PC 2 scores.

### 3.4. Correlation Between E-Tongue and Key Taste Compounds

A negative and positive correlation between key taste compounds is indicated, respectively, by blue and red colors in the upper triangular heat map. The color and type of lines indicate the r and *p* values of the Mantel correlations between molecule levels and E-tongue sensor responses, respectively.

**Figure 5:**
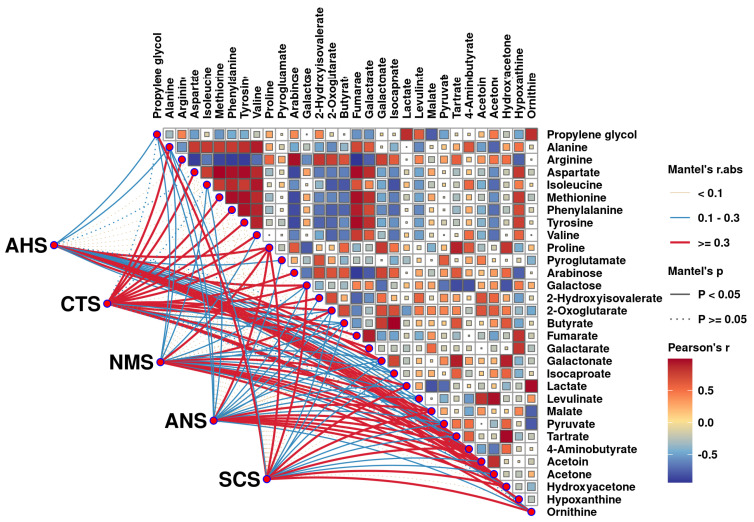
Correlations between key taste compounds quantified by 1H-NMR and E-tongue sensor responses. A negative and positive correlation between key taste compounds is indicated, respectively, by blue and red colors in the upper triangular heat map. The color and type of lines indicate the r and *p* values of Mantel correlations between molecule levels and E-tongue sensor responses, respectively.

To explore the relationship between specific compounds and taste attributes, as well as correlations among taste compounds, a correlation analysis was conducted using E-tongue response values and key taste compounds characterized by ^1^H-NMR, as depicted in Figure 5**.** Aspartate, which is derived from the degradation of nucleic acids, is associated with both a fresh and savory taste [38]. In our study, aspartate exhibited a significant positive correlation with the CTS sensor, which is sensitive to saltiness, highlighting the contribution of aspartate to the overall taste profile of KW. Proline is a sweet amino acid that exhibits a significant positive correlation with ANS sensors, sensitive to sweetness. Furthermore, lactate, levulinate, malate, and pyruvate demonstrated significant positive correlations with the AHS sensor, which is highly sensitive to sourness, underscoring the critical role of organic acids in the acidity and overall sensory attributes of KW.

### 3.5. Correlation Between Key Aroma and Taste Compounds

**Figure 6:**
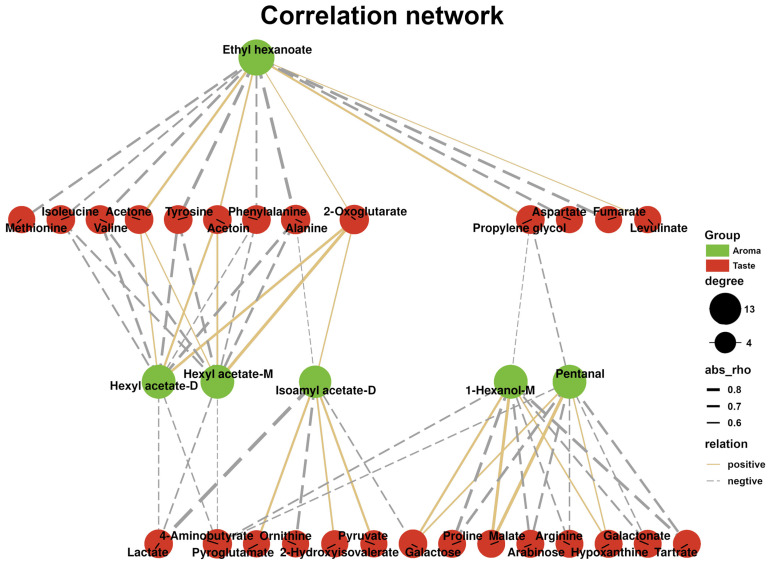
Pearson correlation network between key aroma and key taste compounds in KW. Solid yellow lines represent significant positive correlations, and dashed gray lines represent significant negative correlations (|r| > 0.5). The thicker the line, the stronger the correlation.

Relationships between key aroma and taste compounds in KW were analyzed based on the Pearson correlation coefficients, as shown in Figure 6. These key aroma and taste compounds significantly influence the aroma and flavor profile in KW, thereby affecting its overall quality [39].

Valine showed a negative correlation with hexyl acetate and ethyl hexanoate. Similarly, isoleucine exhibited negative correlations with ethyl hexanoate, hexyl acetate-D, and hexyl acetate-M. These branched-chain amino acids undergo transamination and oxidative decarboxylation reactions during metabolism, potentially influencing the production of butyrate [40]. Moreover, a negative correlation between 1-hexanol-M and proline was found, probably because amino acids could lead to the formation of higher alcohols via the Ehrlich pathway [41].

## 4. Conclusions

This study investigated the effects of the g-IDY addition on the flavor profiles of KW from different kiwifruit cultivars, considering both aroma and taste compounds. E-tongue analysis effectively differentiated the taste characteristics of KW fermented with/without the g-IDY addition. The results indicated that the addition of g-IDY had a negative impact on ester production, while positively influencing the production of many amino acids and organic acids. In detail, the levels of fumarate, galactarate, and lactate were significantly increased in KW produced from green- and yellow-flesh kiwifruits with added g-IDY, while the levels of malate, 2-hydroxyisovalerate, 2-oxoglutarate, butyrate, and galactonate were significantly decreased. It is worth noticing that the flavor of KW fermented by green- and yellow-flesh kiwifruits could be more easily altered by the g-IDY addition, compared to those fermented from red-flesh kiwifruit. This study provides supportive data on the effect of g-IDY on the flavor quality of KW made from kiwifruits with different flesh colors and sheds light on the application of the g-IDY addition in the KW industry. Further investigation is required to explore the impact of different levels of the g-IDY addition on the flavor characteristics of KW, in particular for different flesh colors.

## Figures and Tables

**Table 1:** Information about key aroma compounds.

Compounds	CAS	Peak Intensity		*p* Value	Trend	Peak Intensity		*p* Value	Trend	Peak Intensity		*p* Value	Trend	Odor Threshold Value (mg/Kg)	ROAV					
		GG	GC			RG	RC			YG	YC				GG	GC	RG	RC	YG	YC
Ethyl hexanoate	123-66-0	8.98 × 10^2^ ± 80.8	2.54 × 10^3^ ± 1.75 × 10^2^	0.013	↑	2.81 × 10^3^ ± 1.26 × 10^2^	3.20 × 10^3^ ± 3.46 × 10^2^	0.085	=	2.46 × 10^3^ ± 92.6	1.97 × 10^3^ ± 29.1	0.060	=	0.005	9.70	41.40	34.83	49.45	36.49	35.91
Hexyl acetate-D	142-92-7	1.29 × 10^2^ ± 9.22	7.28 × 10^2^ ± 25.3	0.001	↑	1.13 × 10^2^ ± 17.5	1.07 × 10^2^ ± 31.2	0.577	=	2.63 × 10^2^ ± 7.65	5.85 × 10^2^ ± 1.16 × 10^2^	0.005	↑	0.002	3.48	29.75	3.48	4.04	9.75	26.66
Hexyl acetate-M	142-92-7	2.73 × 10^2^ ± 1.32 × 10	1.43 × 10^3^ ± 14.8	0.001	↑	2.86 × 10^2^ ± 31.4	1.71 × 10^2^ ± 16.5	0.157	↓	8.53 × 10^2^ ± 15.5	1.11 × 10^3^ ± 1.15 × 10^2^	0.007	↑	0.002	7.38	58.24	8.90	6.74	31.67	50.63
Isoamyl acetate-D	123-92-2	1.32 × 10^2^ ± 8.26	1.95 × 10^3^ ± 1.15 × 10^2^	0.001	↑	2.41 × 10^3^ ± 6.01 × 10^2^	8.43 × 10^2^ ± 3.59 × 10^2^	0.152	=	2.87 × 10^3^ ± 41.9	1.86 × 10^3^ ± 50.4	0.010	↓	0.088	0.08	1.80	1.72	0.77	2.42	1.92
1-Hexanol-M	111-27-3	1.18 × 10^4^ ± 2.02 × 10^2^	5.53 × 10^3^ ± 4.21 × 10^2^	0.013	↓	4.12 × 10^3^ ± 6.82 × 10^2^	4.26 × 10^3^ ± 5.18 × 10^2^	0.260	=	5.28 × 10^3^ ± 1.53 × 10^2^	3.85 × 10^3^ ± 1.42 × 10^2^	0.040	↓	0.0056	83.59	80.33	45.19	58.78	69.96	62.44
Pentanal	110-62-3	7.36 × 10^3^ ± 6.06 × 10^2^	1.56 × 10^3^ ± 77.3	0.002	↓	2.68 × 10^2^ ± 1.93	2.62 × 10^2^ ± 83.1	0.924	=	6.72 × 10^2^ ± 24.3	3.95 × 10^2^ ± 14.2	0.023	↓	0.022	18.04	5.79	0.75	0.90	2.26	1.63

Data are displayed as mean  ±  standard deviation (*n* = 5); “↑” represents significant increase, “↓” represents significant decrease, and “=” represents no significant difference (*p* < 0.05).

**Table 2:** Information about key taste compounds.

	GC	GG	*p* Value	Trend	RC	RG	*p* Value	Trend	YC	YG	*p* Value	Trend
**Alcohols and polyols**												
Propylene glycol	1.47 × 10^−3^ ± 6.39 × 10^−5^	3.75 × 10^−4^ ± 1.24 × 10^−5^	<0.01	↓	4.52 × 10^−3^ ± 1.04 × 10^−4^	1.23 × 10^−3^ ± 5.53 × 10^−5^	<0.01	↓	2.31 × 10^−3^ ± 1.29 × 10^−4^	1.32 × 10^−3^ ± 8.65 × 10^−5^	<0.01	↓
**Amino acids, peptides, and analogs**												
Ornithine	7.18 × 10^−4^ ± 1.46 × 10^−4^	5.57 × 10^−4^ ± 7.69 × 10^−5^	0.073	=	3.09 × 10^−3^ ± 2.69 × 10^−4^	2.67 × 10^−4^ ± 7.71 × 10^−5^	<0.01	↓	2.14 × 10^−4^ ± 6.60 × 10^−5^	2.75 × 10^−5^ ± 1.59 × 10^−5^	<0.01	↓
Alanine	4.18 × 10^−4^ ± 2.09 × 10^−5^	1.96 × 10^−3^ ± 2.33 × 10^−5^	<0.01	↑	1.04 × 10^−3^ ± 9.56 × 10^−6^	1.43 × 10^−3^ ± 9.31 × 10^−5^	<0.01	↑	8.94 × 10^−4^ ± 6.04 × 10^−5^	1.04 × 10^−3^ ± 7.36 × 10^−5^	<0.01	↑
4-Aminobutyrate	4.24 × 10^−4^ ± 4.11 × 10^−5^	1.39 × 10^−3^ ± 1.93 × 10^−5^	<0.01	↑	1.43 × 10^−3^ ± 2.57 × 10^−5^	1.95 × 10^−3^ ± 7.75 × 10^−5^	<0.01	↑	1.19 × 10^−3^ ± 6.13 × 10^−5^	1.69 × 10^−3^ ± 1.27 × 10^−4^	<0.01	↑
Arginine	2.80 × 10^−2^ ± 1.27 × 10^−3^	1.97 × 10^−3^ ± 2.05 × 10^−4^	<0.01	↓	2.59 × 10^−2^ ± 9.24 × 10^−4^	3.29 × 10^−2^ ± 2.23 × 10^−3^	0.115	=	3.17 × 10^−2^ ± 2.27 × 10^−3^	2.48 × 10^−2^ ± 1.42 × 10^−3^	<0.01	↓
Aspartate	1.13 × 10^−4^ ± 2.92 × 10^−5^	5.38 × 10^−4^ ± 5.57 × 10^−6^	<0.01	↑	1.64 × 10^−4^ ± 1.59 × 10^−5^	1.77 × 10^−4^ ± 2.39 × 10^−5^	0.060	=	1.76 × 10^−4^ ± 1.22 × 10^−5^	2.35 × 10^−4^ ± 1.80 × 10^−5^	<0.01	↑
Isoleucine	3.86 × 10^−5^ ± 5.65 × 10^−6^	1.28 × 10^−4^ ± 6.48 × 10^−6^	<0.01	↑	8.88 × 10^−5^ ± 5.62 × 10^−6^	7.45 × 10^−5^ ± 6.19 × 10^−6^	<0.01	↓	6.83 × 10^−5^ ± 7.25 × 10^−6^	1.03 × 10^−4^ ± 9.79 × 10^−6^	<0.01	↑
Methionine	6.95 × 10^−5^ ± 4.79 × 10^−6^	2.22 × 10^−4^ ± 2.50 × 10^−6^	<0.01	↑	8.52 × 10^−5^ ± 5.19 × 10^−6^	9.31 × 10^−5^ ± 5.70 × 10^−6^	0.046	↑	9.88 × 10^−5^ ± 7.84 × 10^−6^	1.23 × 10^−4^ ± 7.70 × 10^−6^	<0.01	↑
Phenylalanine	8.26 × 10^−5^ ± 1.45 × 10^−5^	4.10 × 10^−4^ ± 6.76 × 10^−6^	<0.01	↑	1.58 × 10^−4^ ± 7.19 × 10^−6^	1.44 × 10^−4^ ± 8.66 × 10^−6^	<0.01	↓	1.24 × 10^−4^ ± 1.87 × 10^−5^	2.35 × 10^−4^ ± 1.63 × 10^−5^	<0.01	↑
Proline	8.09 × 10^−5^ ± 6.52 × 10^−6^	2.16 × 10^−5^ ± 8.56 × 10^−6^	<0.01	↓	5.79 × 10^−4^ ± 3.04 × 10^−5^	1.16 × 10^−3^ ± 4.45 × 10^−5^	<0.01	↑	3.00 × 10^−4^ ± 2.42 × 10^−5^	6.23 × 10^−6^ ± 2.72 × 10^−6^	<0.01	↓
Pyroglutamate	1.20 × 10^−4^ ± 2.17 × 10^−5^	3.90 × 10^−4^ ± 9.76 × 10^−5^	<0.01	↑	5.31 × 10^−4^ ± 1.71 × 10^−5^	5.87 × 10^−4^ ± 5.55 × 10^−5^	0.097	=	2.43 × 10^−3^ ± 1.39 × 10^−4^	2.73 × 10^−3^ ± 2.12 × 10^−4^	<0.01	↑
Tyrosine	4.40 × 10^−5^ ± 6.97 × 10^−6^	4.46 × 10^−4^ ± 4.98 × 10^−6^	<0.01	↑	1.27 × 10^−4^ ± 8.64 × 10^−6^	1.29 × 10^−4^ ± 9.17 × 10^−6^	0.010	↑	8.45 × 10^−5^ ± 9.40 × 10^−6^	1.34 × 10^−4^ ± 5.94 × 10^−6^	<0.01	↑
Valine	4.47 × 10^−5^ ± 1.18 × 10^−5^	2.51 × 10^−4^ ± 5.27 × 10^−6^	<0.01	↓	1.41 × 10^−4^ ± 7.09 × 10^−6^	1.37 × 10^−4^ ± 1.55 × 10^−5^	<0.01	↓	1.13 × 10^−4^ ± 1.16 × 10^−5^	1.45 × 10^−4^ ± 1.29 × 10^−5^	<0.01	↑
**Carbohydrates and carbohydrate conjugates**												
Arabinose	6.93 × 10^−3^ ± 7.90 × 10^−4^	1.49 × 10^−3^ ± 3.60 × 10^−4^	<0.01	↓	6.91 × 10^−3^ ± 6.48 × 10^−4^	8.89 × 10^−3^ ± 7.75 × 10^−4^	0.223	=	7.28 × 10^−3^ ± 5.00 × 10^−4^	6.99 × 10^−3^ ± 1.57 × 10^−4^	0.05	=
Galactose	5.79 × 10^−3^ ± 1.82 × 10^−4^	6.26 × 10^−3^ ± 3.93 × 10^−4^	0.053	=	4.16 × 10^−3^ ± 3.65 × 10^−4^	2.41 × 10^−4^ ± 7.12 × 10^−5^	<0.01	↓	3.32 × 10^−3^ ± 2.99 × 10^−4^	3.06 × 10^−3^ ± 1.76 × 10^−4^	0.631	=
**Organic acids and derivatives**												
2-Hydroxyisovalerate	5.29 × 10^−5^ ± 8.91 × 10^−6^	6.28 × 10^−6^ ± 2.81 × 10^−6^	<0.01	↓	5.86 × 10^−5^ ± 7.28 × 10^−6^	5.93 × 10^−5^ ± 1.34 × 10^−5^	0.115	=	1.06 × 10^−4^ ± 4.68 × 10^−6^	7.11 × 10^−5^ ± 7.01 × 10^−6^	<0.01	↓
2-Oxoglutarate	7.92 × 10^−4^ ± 5.69 × 10^−5^	2.02 × 10^−4^ ± 2.68 × 10^−5^	<0.01	↓	2.55 × 10^−4^ ± 1.29 × 10^−5^	6.44 × 10^−4^ ± 3.89 × 10^−5^	<0.01	↑	8.19 × 10^−4^ ± 5.95 × 10^−5^	4.92 × 10^−4^ ± 3.13 × 10^−5^	<0.01	↓
Butyrate	2.91 × 10^−4^ ± 2.13 × 10^−5^	7.62 × 10^−5^ ± 1.81 × 10^−6^	<0.01	↓	1.33 × 10^−4^ ± 8.19 × 10^−6^	2.88 × 10^−4^ ± 2.41 × 10^−5^	<0.01	↑	1.60 × 10^−4^ ± 1.13 × 10^−5^	8.30 × 10^−5^ ± 5.85 × 10^−6^	<0.01	↓
Fumarate	3.92 × 10^−5^ ± 5.47 × 10^−6^	4.03 × 10^−4^ ± 5.56 × 10^−6^	<0.01	↑	3.83 × 10^−5^ ± 5.97 × 10^−6^	5.19 × 10^−5^ ± 3.35 × 10^−6^	0.146	=	4.77 × 10^−5^ ± 4.74 × 10^−6^	7.33 × 10^−5^ ± 8.67 × 10^−6^	<0.01	↑
Galactarate	1.12 × 10^−4^ ± 7.06 × 10^−6^	4.00 × 10^−4^ ± 1.30 × 10^−5^	<0.01	↑	4.81 × 10^−5^ ± 3.60 × 10^−6^	1.09 × 10^−4^ ± 6.68 × 10^−6^	<0.01	↑	1.49 × 10^−4^ ± 9.70 × 10^−6^	3.86 × 10^−5^ ± 6.30 × 10^−6^	<0.01	↓
Galactonate	5.13 × 10^−4^ ± 7.87 × 10^−5^	9.49 × 10^−5^ ± 2.99 × 10^−5^	<0.01	↓	3.33 × 10^−4^ ± 2.71 × 10^−5^	1.16 × 10^−3^ ± 1.14 × 10^−4^	<0.01	↑	7.94 × 10^−4^ ± 5.10 × 10^−5^	1.76 × 10^−4^ ± 1.01 × 10^−5^	<0.01	↓
Isocaproate	1.49 × 10^−4^ ± 6.91 × 10^−6^	3.24 × 10^−5^ ± 1.79 × 10^−6^	<0.01	↓	5.62 × 10^−5^ ± 4.49 × 10^−6^	1.34 × 10^−4^ ± 1.31 × 10^−5^	<0.01	↑	7.16 × 10^−5^ ± 7.43 × 10^−6^	3.00 × 10^−5^ ± 3.15 × 10^−7^	<0.01	↓
Lactate	2.26 × 10^−3^ ± 1.17 × 10^−4^	3.95 × 10^−3^ ± 2.38 × 10^−4^	<0.01	↑	2.18 × 10^−2^ ± 1.56 × 10^−3^	1.70 × 10^−3^ ± 1.77 × 10^−4^	<0.01	↓	1.39 × 10^−3^ ± 1.18 × 10^−4^	1.27 × 10^−3^ ± 9.28 × 10^−5^	0.320	=
Levulinate	2.77 × 10^−5^ ± 1.80 × 10^−6^	9.28 × 10^−6^ ± 1.46 × 10^−6^	<0.01	↓	2.71 × 10^−5^ ± 1.05 × 10^−6^	6.85 × 10^−6^ ± 8.91 × 10^−7^	<0.01	↓	3.58 × 10^−5^ ± 2.60 × 10^−6^	6.43 × 10^−6^ ± 1.65 × 10^−6^	<0.01	↓
Malate	3.77 × 10^−2^ ± 9.64 × 10^−3^	3.82 × 10^−2^ ± 2.63 × 10^−4^	0.428	=	6.06 × 10^−4^ ± 2.05 × 10^−4^	2.18 × 10^−2^ ± 1.79 × 10^−3^	<0.01	↑	2.97 × 10^−2^ ± 1.84 × 10^−3^	2.41 × 10^−2^ ± 2.06 × 10^−3^	<0.01	↓
Pyruvate	7.72 × 10^−4^ ± 1.15 × 10^−4^	8.81 × 10^−4^ ± 4.93 × 10^−5^	0.103	=	2.95 × 10^−4^ ± 1.24 × 10^−5^	1.59 × 10^−3^ ± 1.75 × 10^−4^	<0.01	↑	1.26 × 10^−3^ ± 1.82 × 10^−4^	1.82 × 10^−3^ ± 3.21 × 10^−4^	<0.01	↑
Tartrate	7.73 × 10^−6^ ± 1.89 × 10^−6^	3.91 × 10^−6^ ± 4.84 × 10^−7^	0.008	↓	9.08 × 10^−6^ ± 1.53 × 10^−6^	4.33 × 10^−5^ ± 6.21 × 10^−6^	<0.01	↑	1.67 × 10^−5^ ± 1.59 × 10^−6^	6.17 × 10^−6^ ± 4.86 × 10^−7^	<0.01	↓
**Others**												
Acetoin	1.63 × 10^−4^ ± 9.18 × 10^−6^	8.63 × 10^−5^ ± 6.64 × 10^−6^	<0.01	↓	8.74 × 10^−5^ ± 1.03 × 10^−5^	4.58 × 10^−5^ ± 6.11 × 10^−6^	<0.01	↓	2.65 × 10^−4^ ± 1.87 × 10^−5^	9.12 × 10^−5^ ± 8.76 × 10^−6^	<0.01	↓
Acetone	4.28 × 10^−4^ ± 3.83 × 10^−5^	8.31 × 10^−5^ ± 2.87 × 10^−6^	<0.01	↓	2.87 × 10^−4^ ± 1.58 × 10^−5^	9.05 × 10^−5^ ± 7.00 × 10^−6^	<0.01	↓	4.33 × 10^−4^ ± 3.54 × 10^−5^	8.97 × 10^−5^ ± 9.50 × 10^−6^	<0.01	↓
Hydroxyacetone	2.68 × 10^−5^ ± 3.71 × 10^−6^	2.14 × 10^−5^ ± 5.89 × 10^−6^	0.121	↓	5.27 × 10^−6^ ± 2.29 × 10^−6^	1.71 × 10^−4^ ± 2.61 × 10^−5^	<0.01	↑	6.68 × 10^−5^ ± 3.95 × 10^−6^	1.90 × 10^−5^ ± 2.12 × 10^−6^	<0.01	↓
Hypoxanthine	3.01 × 10^−5^ ± 8.31 × 10^−6^	1.02 × 10^−4^ ± 3.76 × 10^−5^	0.011	↑	2.15 × 10^−5^ ± 5.28 × 10^−6^	2.21 × 10^−5^ ± 5.27 × 10^−6^	0.291	=	4.29 × 10^−5^ ± 5.93 × 10^−6^	3.38 × 10^−5^ ± 1.19 × 10^−5^	0.572	=

Data are displayed as mean  ±  standard deviation (n  =  5); “↑” represents significant increase, “↓” represents significant decrease, and “=” represents no significant difference (*p* < 0.05).

## Data Availability

The original contributions presented in this study are included in the article/Supplementary Materials. Further inquiries can be directed to the corresponding author.

## Supplementary Materials

The following supporting information can be downloaded at: https://www.mdpi.com/article/10.3390/foods14101760/s1, Figure S1: Photos of the three kiwis used; Figure S2: The main setting conditions for ^1^H-NMR; Figure S3: ^1^H-NMR spectrum from one KW sample representative of all the registered spectra. The name of each molecule appears over the signal used for its quantification. To ease the reader’s visual inspection, for each portion, a spectrum with a convenient signal-to-noise ratio has been selected; Figures S4–S16: Pictorial description of the molecules’ assignment and quantification procedure by Chenomx software (v.10.1). Upper panel—portions of the spectra in white-washed mode. Lower panel—One representative registered spectrum (black line) superimposed to the signals simulated by Chenomx software (red line) for each of the molecules listed. The black and red dashed lines evidence the signals used for quantification purposes; Table S1: Information of all aroma compounds identified by GC-IMS; Table S2: Information of all taste compounds identified by ^1^H-NMR.
