# Supplementary material for: Effects of Glutathione-Enriched Inactive Dry Yeast on the Flavor Profile of Kiwi Wine
Source: Foods. 2025 May 15;14(10):1760. doi: 10.3390/foods14101760 (PMC12111175; doi:10.3390/foods14101760)
Supplement: Supplementary file 1 [file foods-14-01760-s001.zip › foods-3612970-supplementary.pdf]

Effect of glutathione-enriched inactive dry yeast on the flavor profile of kiwi wine

Zhibo Yang[1,2,†], Chuan Song[3,†], Qiuyu Lan[4], Bin Hu[2], Zonghua Ao[3], Suyi Zhang[3], Junni Tang[1], Xin Du[5], Chenglin Zhu[1,*], Luca Laghi[4]

Supporting Materials

Relative odor activity value calculation method

The relative odor activity value (ROAV) of each flavor compound was calculated using the following formula:

$$ROAV = 100 \times (C\%_X/C\%_{stan}) \times (T_{stan}/T_X)$$

Where $ROAV_X$ represents the relative odor activity value of a flavor compound, $C\%_X$ denotes its relative content, and T_X indicates its threshold. $C\%_{stan}$ and T_{stan} denote, respectively, the relative content and threshold of flavor compounds that contribute most significantly to the overall flavor. The relative content of each flavor compound was determined using the peak area normalization method.

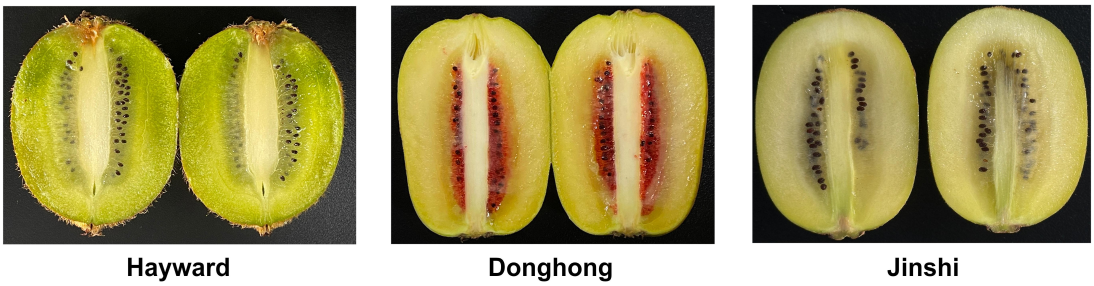

Figure S1. Photos of the three kiwis used.

[*] **Correspondence:** Chenglin Zhu, chenglin.zhu@swun.edu.cn, Tel.: +86-028-85928478

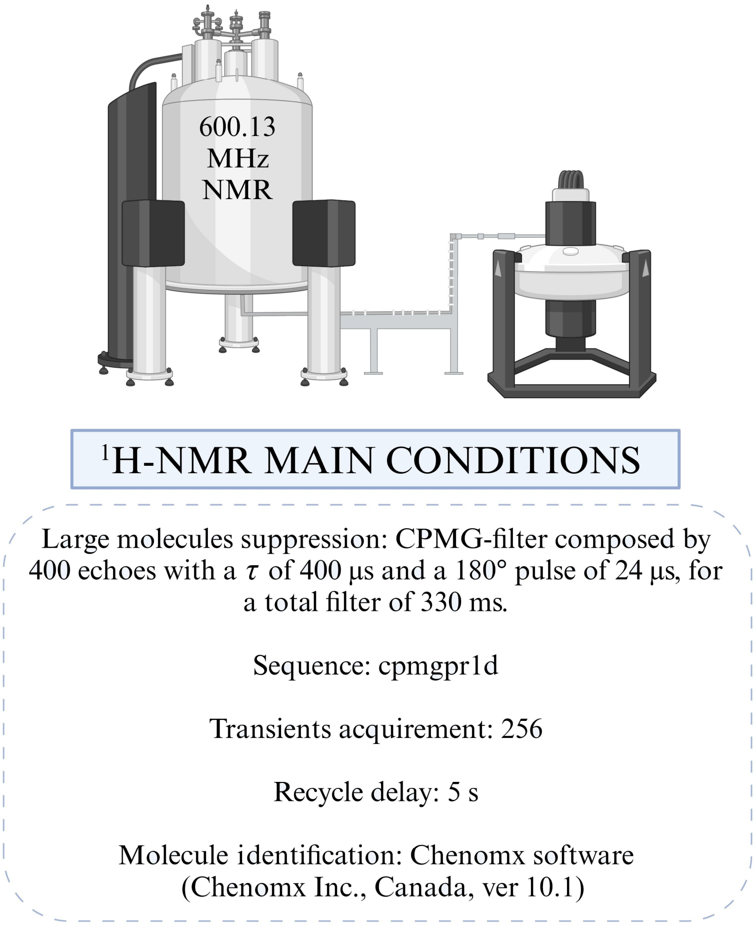

Figure S2. The main setting conditions for ^{1}H-NMR.

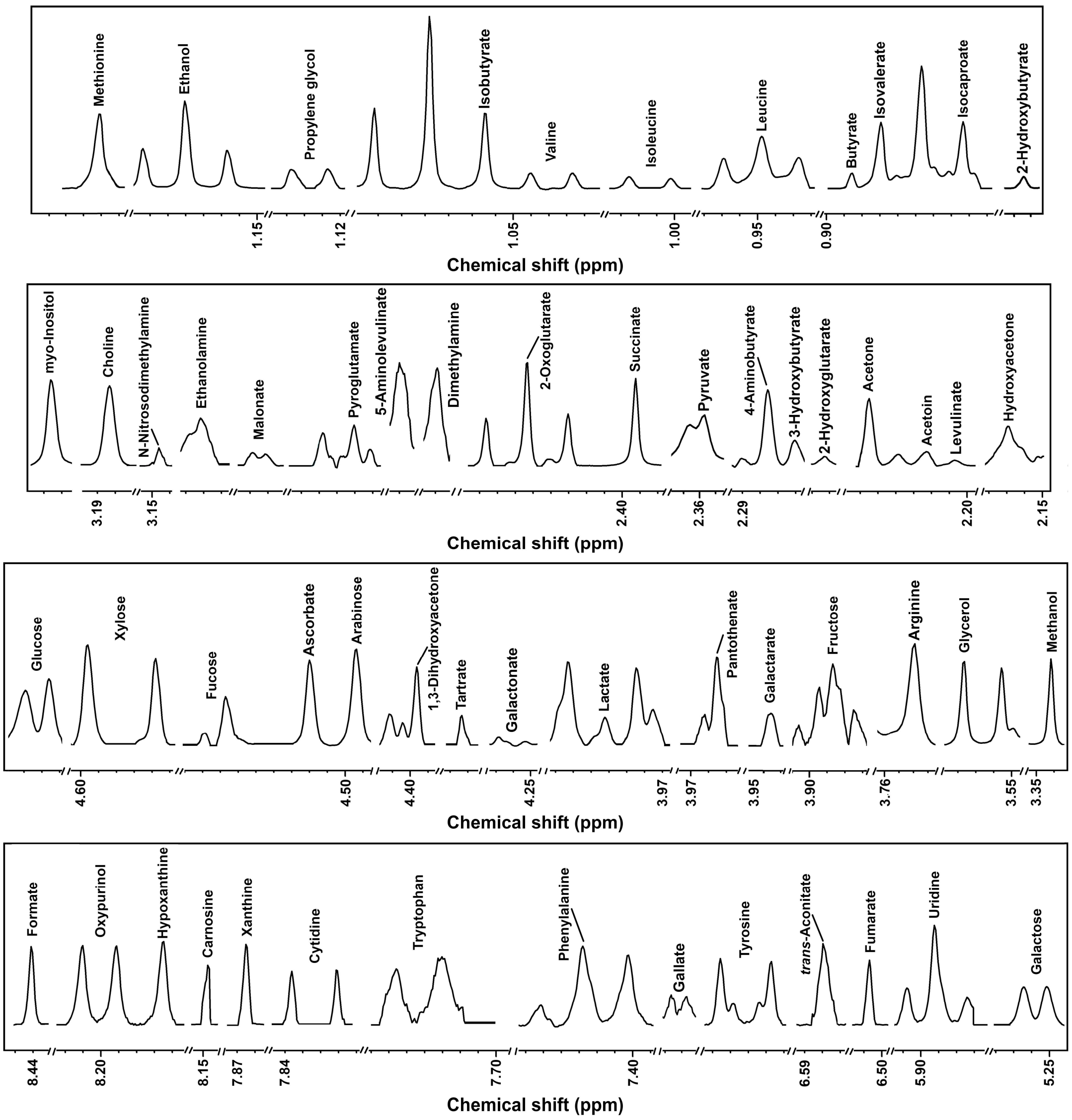

Figure S3. ^{1}H-NMR spectrum from one KW sample representative of all the registered spectra. The name of each molecule appears over the signal used for its quantification. To ease the reader's visual inspection, for each portion a spectrum with a convenient signal-to-noise ratio has been selected.

Figure S4-S16. Pictorial description of the molecules' assignment and quantification procedure by Chenomx software. Upper panel - portions of the spectra in white-washed mode. Lower panel – One representative registered spectrum (black line) superimposed to the signals simulated by Chenomx software (red line) for each of the molecules listed. The black and red dashed lines evidence the signals used for quantification purposes.

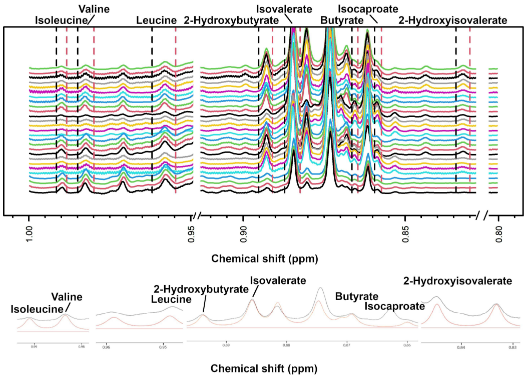

Figure S4

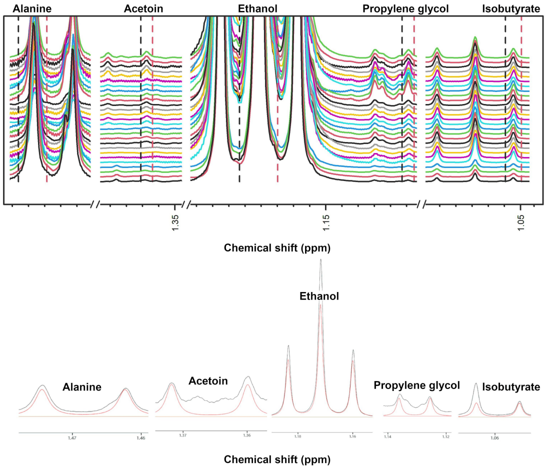

Figure S5

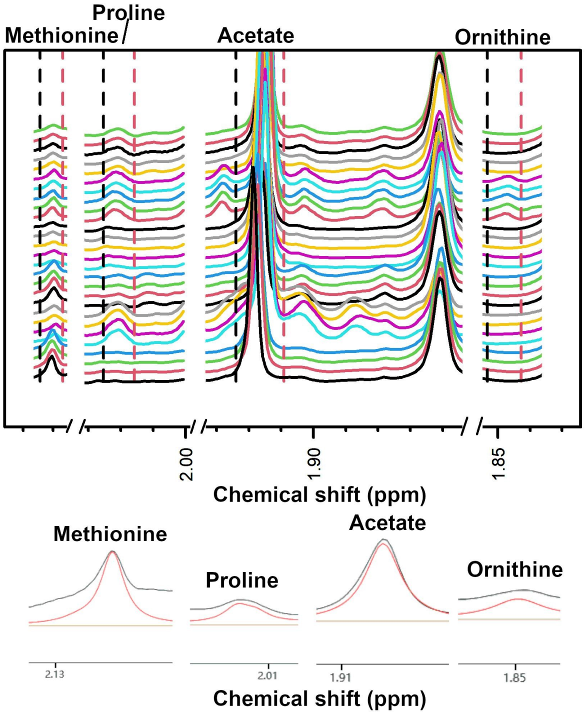

Figure S6

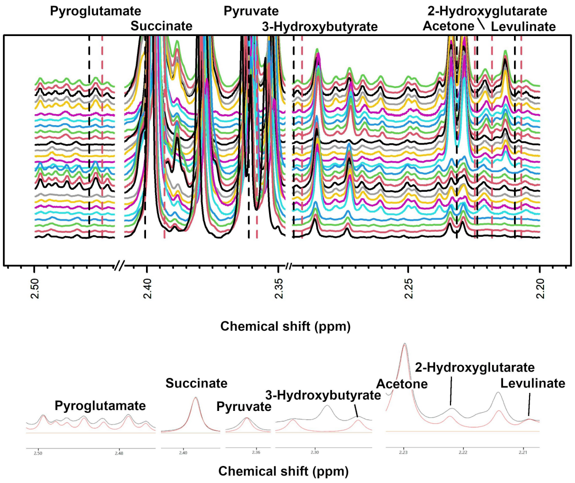

Figure S7

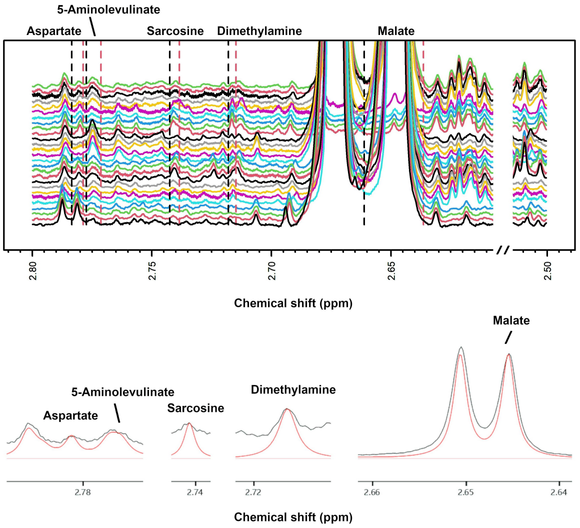

Figure S8

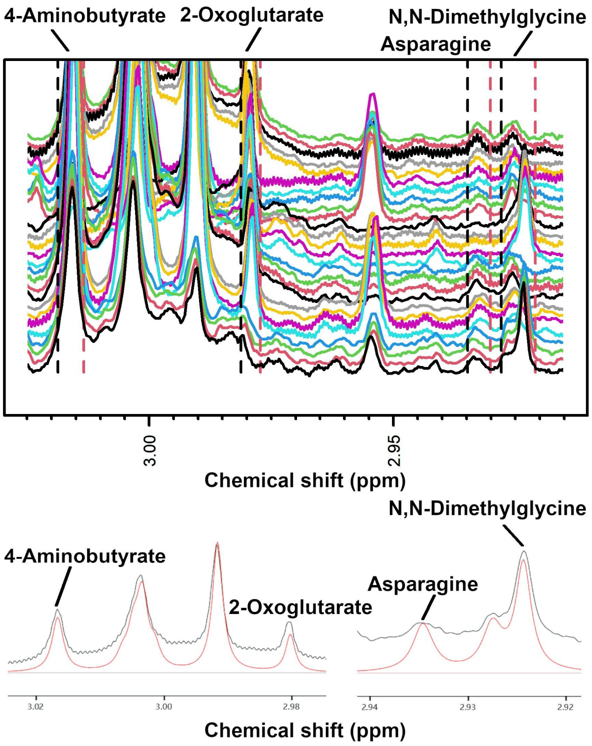

Figure S9

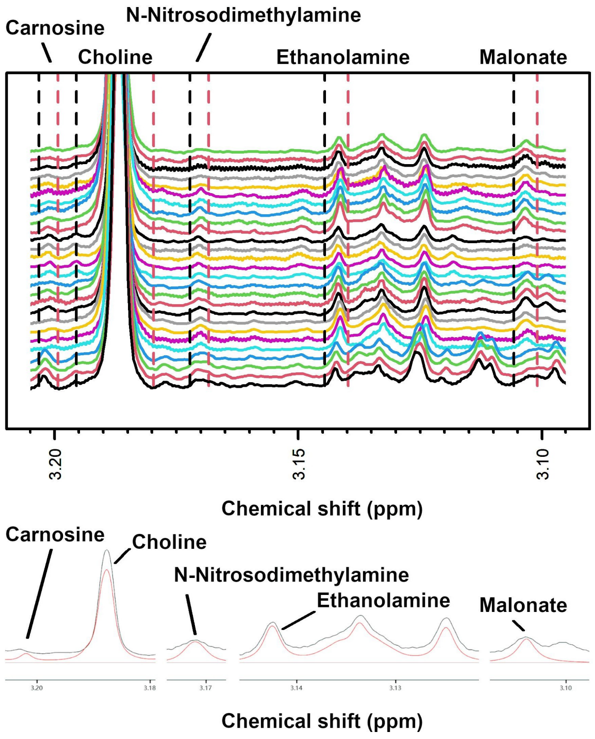

Figure S10

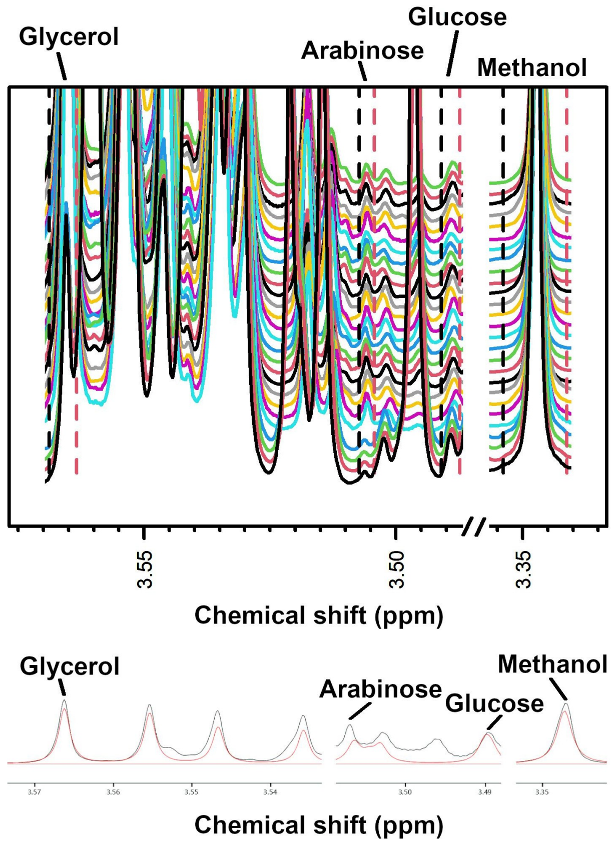

Figure S11

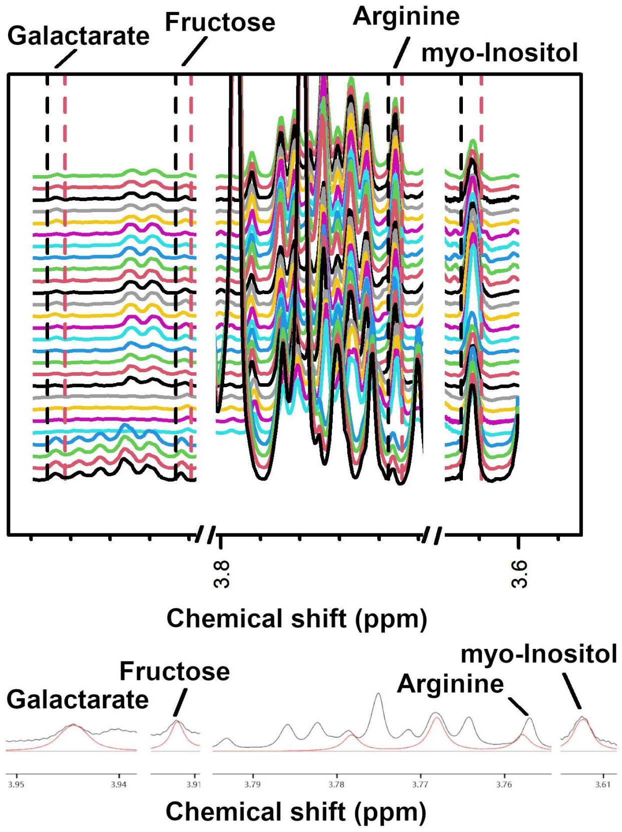

Figure S12

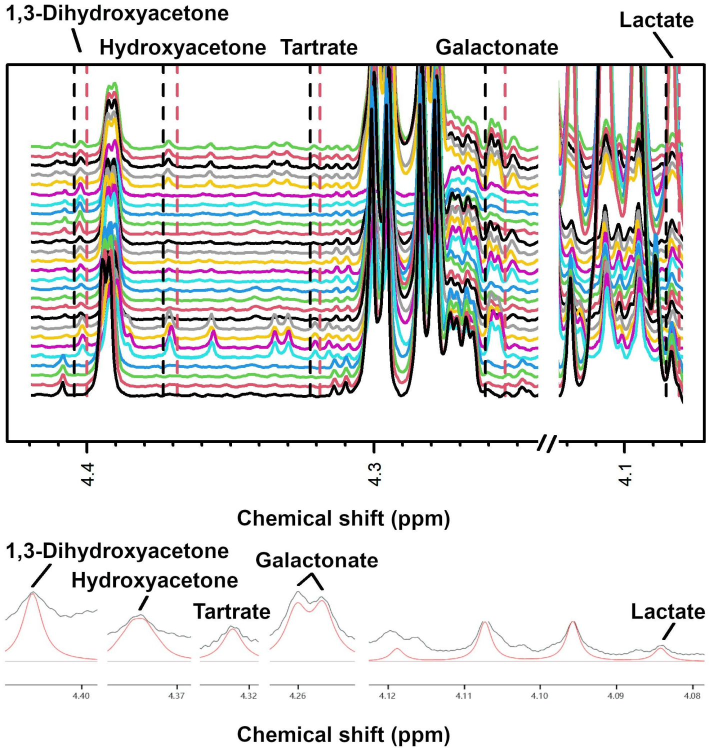

Figure S13

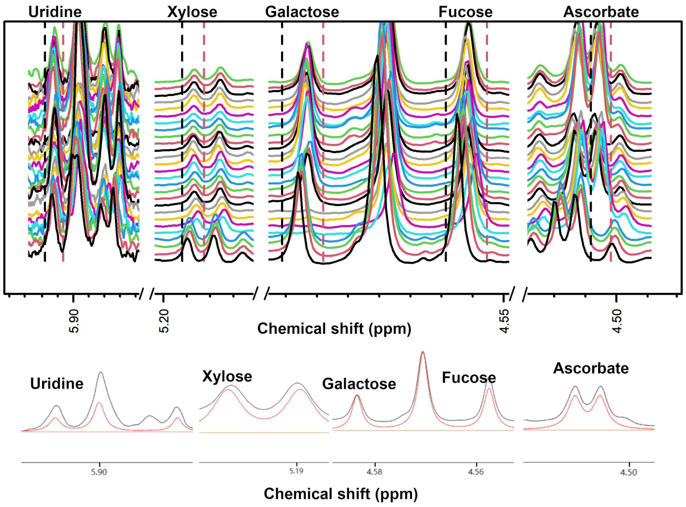

Figure S14

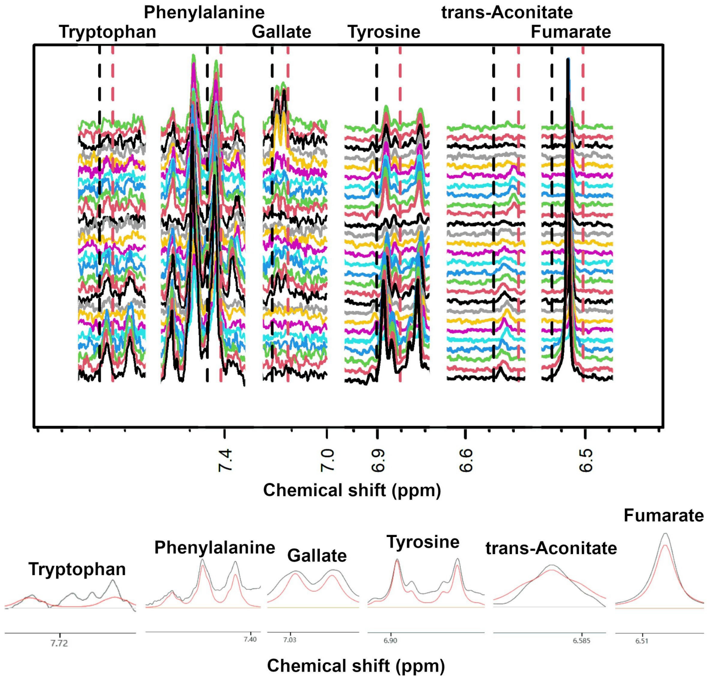

Figure S15

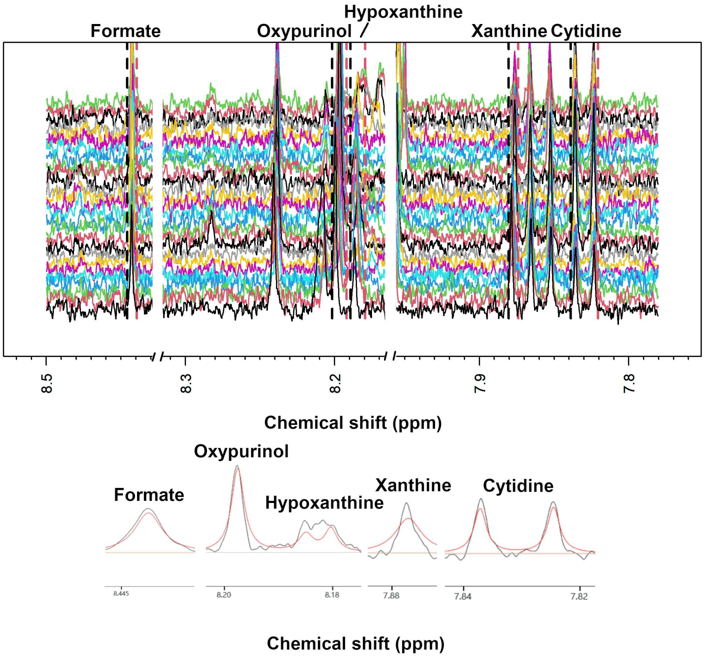

Figure S16

Table S1. Information of all aroma compounds identified by GC-IMS

Compounds	CAS	Peak Intensity		p Value	Trend	Peak Intensity		p Value	Trend	Peak Intensity		p Value	Trend	Threshold value	ROAV					
		GG	GC			RG	RC			YG	YC				GG	GC	RG	RC	YG	YC
Esters																				
cis-3-Hexenyl acetate	3681-71-8	$1.30\times10^{2}\pm1.34\times10$	$2.70\times10^{2}\pm2.59\times10$	0.020	↑	$1.49\times10^{2}\pm3.27\times10$	$2.04\times10^{2}\pm2.22\times10$	0.150	=	$1.80\times10^{2}\pm1.19\times10$	$2.07\times10^{2}\pm1.01\times10$	0.082	=	0.056	0.1	0.3	0.1	0.2	0.2	0.3
Ethyl acetate-D	141-78-6	$9.30\times10^{3}\pm1.46\times10^{3}$	$6.14\times10^{3}\pm3.60\times10^{2}$	0.048	↓	$8.03\times10^{3}\pm7.57\times10^{2}$	$6.40\times10^{3}\pm3.66\times10^{2}$	0.361	=	$6.73\times10^{3}\pm1.93\times10^{2}$	$5.50\times10^{3}\pm1.69\times10^{2}$	0.049	↓	0.005	100.0	100.0	100.0	100.0	100.0	100.0
Ethyl acetate-M	141-78-6	$1.75\times10^{2}\pm8.35\times10$	$1.36\times10^{2}\pm7.00$	0.909	=	$1.20\times10^{2}\pm5.58\times10$	$7.13\times10\pm1.59\times10$	0.446	=	$1.16\times10^{2}\pm1.55\times10$	$1.06\times10^{2}\pm8.53$	0.443	=	0.005	1.8	2.2	1.5	1.1	1.7	1.9
Ethyl butyrate-D	105-54-4	$6.15\times10^{3}\pm1.24\times10^{3}$	$5.05\times10^{3}\pm2.17\times10^{2}$	0.239	=	$7.99\times10^{2}\pm3.42\times10^{2}$	$3.27\times10^{2}\pm8.27\times10$	0.165	=	$1.57\times10^{3}\pm1.07\times10^{2}$	$1.72\times10^{3}\pm1.06\times10^{2}$	0.062	=	0.02	16.5	20.5	2.5	1.3	5.8	7.8
Ethyl butyrate-M	105-54-4	$3.80\times10^{3}\pm5.20\times10^{2}$	$1.77\times10^{3}\pm1.05\times10^{2}$	0.014	↓	$2.17\times10^{3}\pm4.44\times10^{2}$	$1.78\times10^{3}\pm7.30\times10$	0.647	=	$2.12\times10^{3}\pm1.01\times10^{2}$	$1.76\times10^{3}\pm1.09\times10^{2}$	0.289	=	0.02	10.2	7.2	6.7	6.9	7.8	8.0
Ethyl heptanoate	106-30-9	$2.02\times10^{3}\pm3.45\times10^{2}$	$3.75\times10^{3}\pm3.09\times10^{2}$	0.073	=	$2.50\times10^{3}\pm2.75\times10^{2}$	$4.14\times10^{3}\pm5.17\times10^{2}$	0.059	=	$3.43\times10^{3}\pm6.90\times10$	$3.28\times10^{3}\pm9.98\times10$	0.051	=	0.22	0.5	1.3	0.6	1.4	1.1	1.3
Ethyl hexanoate	123-66-0	$8.98\times10^{2}\pm8.08\times10$	$2.54\times10^{3}\pm1.75\times10^{2}$	0.013	↑	$2.81\times10^{3}\pm1.26\times10^{2}$	$3.20\times10^{3}\pm3.46\times10^{2}$	0.085	=	$2.46\times10^{3}\pm9.26\times10$	$1.97\times10^{3}\pm2.91\times10$	0.060	=	0.005	9.7	41.4	34.8	49.4	36.4	35.9
Ethyl isobutyrate	97-62-1	$4.66\times10^{2}\pm1.78\times10^{2}$	$2.20\times10^{2}\pm3.61\times10$	0.048	↓	$2.24\times10^{2}\pm1.20\times10^{2}$	$4.01\times10^{2}\pm1.07\times10^{2}$	0.157	=	$1.33\times10^{2}\pm1.61\times10$	$2.34\times10^{2}\pm1.89\times10$	0.032	↑	0.015	1.7	1.1	0.9	2.0	0.6	1.4
Ethyl isovalerate	108-64-5	$1.91\times10^{3}\pm1.78\times10^{2}$	$2.45\times10^{3}\pm1.83\times10^{2}$	0.123	=	$3.20\times10^{3}\pm2.87\times10^{2}$	$3.26\times10^{3}\pm3.56\times10^{2}$	0.106	=	$2.23\times10^{3}\pm5.05\times10$	$1.87\times10^{3}\pm6.55\times10$	0.181	=	0.03	3.4	6.6	6.6	8.3	5.5	5.6
Ethyl octanoate-D	106-32-1	$1.66\times10^{2}\pm2.71\times10$	$1.77\times10^{2}\pm2.72\times10$	0.536	=	$3.47\times10^{2}\pm8.47\times10$	$1.71\times10^{2}\pm1.45\times10$	0.063	=	$3.24\times10^{2}\pm6.88\times10$	$2.13\times10^{2}\pm8.71\times10$	0.389	=	0.005	1.8	2.9	4.3	2.6	4.8	3.8
Ethyl octanoate-M	106-32-1	$2.16\times10^{2}\pm3.40\times10$	$8.29\times10^{2}\pm9.03\times10$	0.013	↑	$1.32\times10^{3}\pm2.94\times10^{2}$	$5.87\times10^{2}\pm2.20\times10^{2}$	0.189	=	$1.26\times10^{3}\pm1.28\times10^{2}$	$7.83\times10^{2}\pm2.50\times10^{2}$	0.294	=	0.005	2.3	13.5	16.6	9.5	18.8	14.3
Ethyl pentanoate	539-82-2	$1.78\times10^{2}\pm2.26\times10$	$3.32\times10^{2}\pm2.14\times10$	0.035	↑	$3.29\times10^{2}\pm2.36\times10$	$3.27\times10^{2}\pm4.27\times10$	0.211	=	$2.31\times10^{2}\pm9.05$	$2.65\times10^{2}\pm7.99$	0.042	↑	0.0049	2.0	5.5	4.1	5.2	3.5	4.9
Hexyl acetate-D	142-92-7	$1.29\times10^{2}\pm9.22$	$7.28\times10^{2}\pm2.53\times10$	0.001	↑	$1.13\times10^{2}\pm1.75\times10$	$1.07\times10^{2}\pm3.12\times10$	0.577	=	$2.63\times10^{2}\pm7.65$	$5.85\times10^{2}\pm1.16\times10^{2}$	0.005	↑	0.002	3.5	29.8	3.5	4.0	9.8	26.7
Hexyl acetate-M	142-92-7	$2.73\times10^{2}\pm1.32\times10$	$1.43\times10^{3}\pm1.48\times10$	<0.01	↑	$2.86\times10^{2}\pm3.14\times10$	$1.71\times10^{2}\pm1.65\times10$	0.157	=	$8.53\times10^{2}\pm1.55\times10$	$1.11\times10^{3}\pm1.15\times10^{2}$	0.007	↑	0.002	7.4	58.2	8.9	6.7	31.7	50.6
Isoamyl acetate-D	123-92-2	$1.32\times10^{2}\pm8.26$	$1.95\times10^{3}\pm1.15\times10^{2}$	<0.01	↑	$2.41\times10^{3}\pm6.01\times10^{2}$	$8.43\times10^{2}\pm3.59\times10^{2}$	0.152	=	$2.87\times10^{3}\pm4.19\times10$	$1.86\times10^{3}\pm5.04\times10$	0.010	↓	0.088	0.1	1.8	1.7	0.8	2.4	1.9
Isoamyl acetate-M	123-92-2	$5.83\times10^{2}\pm7.28\times10$	$1.06\times10^{3}\pm4.89\times10$	0.042	↑	$1.51\times10^{3}\pm2.33\times10^{2}$	$6.91\times10^{2}\pm1.29\times10^{2}$	0.117	=	$1.33\times10^{3}\pm1.01\times10^{2}$	$8.00\times10^{2}\pm3.26\times10$	0.004	↓	0.088	0.4	1.0	1.1	0.6	1.1	0.8
Isobutyl acetate-D	110-19-0	$1.73\times10^{2}\pm3.16\times10$	$1.64\times10^{2}\pm9.29$	0.830	=	$5.29\times10^{2}\pm2.05\times10^{2}$	$2.54\times10^{2}\pm3.35\times10$	0.153	=	$3.59\times10^{2}\pm9.62$	$1.96\times10^{2}\pm7.98$	0.023	↓	1.6	<0.01	<0.01	<0.1	<0.1	<0.1	<0.1
Isobutyl acetate-M	110-19-0	$4.51\times10^{2}\pm3.35\times10$	$1.81\times10^{2}\pm1.87\times10$	0.015	↓	$4.85\times10^{2}\pm2.91\times10$	$3.26\times10^{2}\pm1.40\times10^{2}$	0.231	=	$2.78\times10^{2}\pm2.20\times10$	$1.54\times10^{2}\pm7.96$	0.007	↓	1.6	<0.1	<0.01	<0.1	<0.1	<0.1	<0.01
Methyl acetate	79-20-	$1.76\times10^{3}\pm8.09\times10^{2}$	$1.11\times10^{3}\pm5.66\times10$	0.784	=	$1.42\times10^{3}\pm7.55\times10^{2}$	$3.61\times10^{2}\pm1.50\times10^{2}$	0.114	=	$7.29\times10^{2}\pm2.38\times10$	$8.32\times10^{2}\pm1.41\times10$	0.006	↑	0.003	31.3	30.0	30.1	9.7	18.0	25.2

	9																			
Methyl isobutyrate	547-63-7	$4.5\times10^{2}\pm9.69\times10$	$2.23\times10^{2}\pm1.21\times10$	0.011	↓	$3.85\times10^{2}\pm1.25\times10^{2}$	$6.53\times10^{2}\pm1.37\times10^{2}$	0.136	=	$5.67\times10^{2}\pm8.90$	$8.25\times10^{2}\pm1.82\times10$	0.001	↑	0.007	3.5	2.6	3.5	7.4	6.0	10.7
Propyl acetate	109-60-4	$5.35\times10\pm9.20$	$5.41\times10\pm3.94$	0.602	=	$1.15\times10^{2}\pm9.55$	$9.47\times10\pm1.76\times10$	0.315	=	$8.64\times10\pm4.42$	$7.12\times10\pm1.54$	0.119	=	2.7	<0.01	<0.01	<0.01	<0.01	<0.01	<0.01
Propyl butyrate	105-66-8	$2.04\times10^{2}\pm4.22\times10$	$3.58\times10^{2}\pm3.52\times10$	0.098	=	$1.04\times10^{3}\pm1.15\times10^{2}$	$7.83\times10^{2}\pm1.18\times10^{2}$	0.174	=	$3.78\times10^{2}\pm2.24\times10$	$4.82\times10^{2}\pm1.46\times10$	0.032	↑	0.124	0.1	0.2	0.5	0.5	0.2	0.4
															0	0	0	0	0	0
Alcohols																				
1-Hexanol-D	111-27-3	$5.34\times10^{3}\pm9.91\times10^{2}$	$6.39\times10^{3}\pm4.79\times10^{2}$	0.293	=	$1.55\times10^{3}\pm3.39\times10^{2}$	$2.76\times10^{3}\pm2.52\times10^{2}$	0.076	=	$4.30\times10^{3}\pm6.95\times10$	$4.46\times10^{3}\pm4.74\times10$	0.040	↑	0.0056	51.8	92.9	17.0	38.2	57.0	72.4
1-Hexanol-M	111-27-3	$1.18\times10+4\pm2.02\times10^{2}$	$5.53\times10^{3}\pm4.21\times10^{2}$	0.013	↑	$4.12\times10^{3}\pm6.82\times10^{2}$	$4.26\times10^{3}\pm5.18\times10^{2}$	0.260	=	$5.28\times10^{3}\pm1.53\times10^{2}$	$3.85\times10^{3}\pm1.42\times10^{2}$	0.040	↓	0.0056	83.6	80.3	45.2	58.8	70.0	62.4
Isobutanol-D	78-83-1	$4.09\times10^{3}\pm5.51\times10^{2}$	$4.23\times10^{3}\pm3.05\times10^{2}$	0.440	=	$5.69\times10^{3}\pm4.81\times10^{2}$	$5.28\times10^{3}\pm5.20\times10^{2}$	0.301	=	$3.42\times10^{3}\pm8.88\times10$	$3.01\times10^{3}\pm9.82\times10$	0.123	=	7	<0.1	<0.1	0.1	0.1	<0.1	<0.1
Isobutanol-M	78-83-1	$4.62\times10^{3}\pm7.94\times10^{2}$	$1.97\times10^{3}\pm1.32\times10^{2}$	0.014	↓	$2.77\times10^{3}\pm1.64\times10^{2}$	$2.77\times10^{3}\pm3.68\times10^{2}$	0.039	↑	$2.13\times10^{3}\pm9.53\times10$	$1.73\times10^{3}\pm2.48\times10$	0.158	=	7	<0.1	<0.1	<0.1	<0.1	<0.1	<0.1
Isopropanol	67-63-0	$4.56\times10^{2}\pm7.56\times10$	$6.05\times10^{2}\pm5.11\times10$	0.162	=	$6.81\times10^{2}\pm3.34\times10$	$9.41\times10^{2}\pm8.24\times10$	0.010	↑	$7.03\times10^{2}\pm1.71\times10$	$7.78\times10^{2}\pm2.88\times10$	0.027	↑	0.08	0.3	0.6	0.5	0.9	0.7	0.9
trans-3-Hexen-1-ol-M	928-97-2	$2.46\times10^{3}\pm1.46\times10^{2}$	$6.85\times10^{2}\pm5.12\times10$	0.005	↓	$8.23\times10^{2}\pm1.80\times10^{2}$	$7.33\times10^{2}\pm1.21\times10^{2}$	0.575	=	$6.95\times10^{2}\pm3.43\times10$	$4.70\times10^{2}\pm2.41\times10$	0.043	↓	1	0.1	0.1	0.1	0.1	0.1	<0.1
trans-3-Hexen-2-ol-D	928-97-2	$7.69\times10^{2}\pm1.76\times10^{2}$	$2.97\times10^{2}\pm1.77\times10$	0.008	↓	$2.21\times10^{2}\pm6.38\times10$	$2.39\times10^{2}\pm1.67\times10$	0.305	=	$2.51\times10^{2}\pm2.24\times10$	$1.97\times10^{2}\pm2.70\times10$	0.377	=	1	<0.1	<0.1	<0.1	<0.1	<0.1	<0.1
Ketones															0	0	0	0	0	0
2,6-Dimethylheptan-4-one	108-83-8	$6.48\times10^{2}\pm2.68\times10^{2}$	$6.16\times10^{2}\pm5.91\times10$	0.526	=	$7.03\times10^{2}\pm3.62\times10^{2}$	$6.64\times10^{2}\pm8.70\times10$	0.465	=	$4.77\times10^{2}\pm4.28$	$5.13\times10^{2}\pm1.54\times10$	0.010	↑	0	0	0	0	0	0	0
2,6-Dimethylheptan-4-one-M	108-83-8	$5.06\times10^{3}\pm3.51\times10^{2}$	$5.31\times10^{3}\pm4.53\times10^{2}$	0.412	=	$8.67\times10^{3}\pm6.18\times10^{2}$	$9.43\times10^{3}\pm1.02\times10^{3}$	0.150	=	$5.53\times10^{3}\pm6.43\times10$	$4.75\times10^{3}\pm1.33\times10^{2}$	0.451	=	0	0	0	0	0	0	0
2,6-Dimethylheptan-4-one-D	108-83-8	$4.71\times10^{2}\pm3.16\times10^{2}$	$9.72\times10^{2}\pm1.70\times10$	0.163	=	$1.08\times10^{3}\pm3.78\times10$	$2.46\times10^{2}\pm1.27\times10$	0.003	↓	$2.71\times10^{3}\pm2.82\times10$	$1.04\times10^{3}\pm1.80\times10^{2}$	0.051	=	0	0	0	0	0	0	0
2-Butanone	78-93-3	$6.15\times10^{2}\pm5.74\times10$	$6.67\times10^{2}\pm4.58\times10$	0.330	=	$1.03\times10^{3}\pm8.73\times10$	$1.36\times10^{3}\pm5.33\times10$	0.031	↑	$9.59\times10^{2}\pm2.57\times10$	$9.54\times10^{2}\pm2.97\times10$	0.095	=	0	0	0	0	0	0	0
2-Pentanone	107-87-9	$1.93\times10^{2}\pm3.87\times10$	$1.94\times10^{2}\pm1.16\times10$	0.529	=	$1.90\times10^{2}\pm7.26\times10$	$1.75\times10^{2}\pm4.29\times10$	0.642	=	$2.68\times10^{2}\pm9.63$	$3.52\times10^{2}\pm1.22\times10$	0.003	↑	1.38	<0.01	<0.1	<0.01	<0.1	<0.1	<0.1
Acetone	67-64-1	$1.01\times10^{3}\pm7.29\times10^{2}$	$9.54\times10^{2}\pm9.40\times10$	0.428	=	$1.60\times10^{3}\pm8.96\times10^{2}$	$2.82\times10^{3}\pm1.51\times10^{3}$	0.185	=	$1.08\times10^{3}\pm1.04\times10$	$8.74\times10^{2}\pm4.12\times10$	0.159	=	0.08	0.7	1.0	1.2	2.6	1.0	1.0
															0	0	0	0	0	0
Aldehydes																				
2-Methylbutanal	96-17-3	$1.38\times10^{2}\pm2.94\times10$	$6.83\times10\pm3.48$	0.010	↓	$9.80\times0\pm2.86\times10$	$9.17\times10\pm1.61\times10$	0.617	=	$1.22\times10^{2}\pm1.01$	$1.42\times10^{2}\pm7.41$	0.003	↑	0.0044	1.7	1.3	1.4	1.7	2.1	2.9
Butanal	123-72-8	$5.36\times10\pm9.87$	$5.85\times10\pm5.48$	0.452	=	$4.10\times10\pm4.80$	$6.76\times10\pm5.52$	0.045	↑	$4.51\times10\pm1.58$	$3.60\times10\pm1.90$	0.104	=	0.009	0.3	0.5	0.3	0.6	0.4	0.4

Pentanal	110-62-3	$7.36\times10^{3}\pm6.06\times10^{2}$	$1.56\times10^{3}\pm7.73\times10$	0.002	↓	$2.68\times10^{2}\pm1.93$	$2.62\times10^{2}\pm8.31\times10$	0.924	=	$6.72\times10^{2}\pm2.43\times10$	$3.95\times10^{2}\pm1.42\times10$	0.023	↓	0.022	18.0	5.8	0.8	0.9	2.3	1.6
Others															0	0	0	0	0	0
2,5-Dimethylfuran	625-86-5	$4.76\times10^{2}\pm3.07\times10$	$5.55\times10^{2}\pm4.13\times10$	0.139	=	$1.30\times10^{2}\pm1.59\times10$	$3.01\times10^{2}\pm2.18\times10$	0.007	↑	$5.42\times10^{2}\pm1.22\times10$	$7.26\times10^{2}\pm1.66\times10$	0.003	↑	0	0	0	0	0	0	0
2-Butylfuran	4466-24-4	$3.39\times10^{2}\pm1.16\times10$	$1.75\times10^{2}\pm2.21\times10$	0.030	↓	$4.59\times10^{2}\pm7.39\times10$	$3.84\times10^{2}\pm6.28\times10$	0.865	=	$1.85\times10^{2}\pm1.86\times10$	$2.07\times10^{2}\pm5.18$	0.115	=	0.005	3.7	2.8	5.6	5.9	2.7	3.8
2-Pentylfuran	3777-69-3	$1.50\times10^{3}\pm1.85\times10^{2}$	$6.21\times10^{2}\pm5.20\times10$	0.016	↓	$6.92\times10^{2}\pm1.12\times10^{2}$	$6.64\times10^{2}\pm8.43\times10$	0.362	=	$4.51\times10^{2}\pm1.76\times10$	$4.41\times10^{2}\pm1.36\times10$	0.082	=	0.006	13.5	8.4	7.1	8.5	5.6	6.7
alpha-Terpinolene	586-62-9	$3.26\times10^{2}\pm6.89\times10$	$3.14\times10^{2}\pm4.27\times10$	0.799	=	$2.21\times10^{2}\pm5.20\times10$	$9.87\times10^{2}\pm1.29\times10^{2}$	0.010	↑	$2.06\times10^{2}\pm8.28$	$8.65\times10^{2}\pm3.93\times10$	0.001	↑	0.2	0.1	0.1	0.1	0.4	0.1	0.4

Data are displayed as mean ± standard deviation (n = 5).

* "↑" represents significant increase, "↓" represents significant decrease, "=" represents no significant difference ($p < 0.05$).

The threshold value of volatile compounds primary reference prior studies (Wang et al., 2022; Liu eta al., 2022; Yang et al., 2022; Lan et al., 2022).

Table S2. Information of all taste compounds identified by ^{1}H-NMR.

Compounds	GC	GG	*p* Value	Trend	RC	RG	*p* Value	Trend	YC	YG	*p* Value	Trend
Alcohols and polyols												
Ethanol	$1.13 \pm 6.52\times10^{-2}$	$3.81\times10^{-1} \pm 3.69\times10^{-3}$	<0.01	↓	$9.19\times10^{-1} \pm 2.85\times10^{-2}$	$1.25 \pm 9.60\times10^{-2}$	<0.01	↑	$1.06 \pm 6.72\times10^{-2}$	$9.18\times10^{-1} \pm 6.67\times10^{-2}$	0.02	↓
Glycerol	$5.11\times10^{-2} \pm 3.18\times10^{-3}$	$1.85\times10^{-2} \pm 5.34\times10^{-4}$	<0.01	↓	$5.19\times10^{-2} \pm 8.25\times10^{-4}$	$5.34\times10^{-2} \pm 2.84\times10^{-3}$	<0.01	↑	$6.91\times10^{-2} \pm 3.82\times10^{-3}$	$5.11\times10^{-2} \pm 4.02\times10^{-3}$	<0.01	↓
Methanol	$7.80\times10^{-3} \pm 4.36\times10^{-4}$	$1.12\times10^{-2} \pm 6.16\times10^{-5}$	<0.01	↑	$3.95\times10^{-3} \pm 1.24\times10^{-4}$	$1.02\times10^{-2} \pm 7.89\times10^{-4}$	<0.01	↑	$7.30\times10^{-3} \pm 5.03\times10^{-4}$	$5.23\times10^{-3} \pm 4.05\times10^{-4}$	<0.01	↓
myo-Inositol	$1.28\times10^{-2} \pm 7.49\times10^{-4}$	$1.45\times10^{-2} \pm 8.71\times10^{-5}$	<0.01	↑	$6.17\times10^{-3} \pm 1.20\times10^{-4}$	$5.72\times10^{-3} \pm 2.21\times10^{-4}$	<0.01	↓	$4.80\times10^{-3} \pm 2.09\times10^{-4}$	$4.34\times10^{-3} \pm 3.37\times10^{-4}$	0.63	=
Propylene glycol	$1.47\times10^{-3} \pm 6.39\times10^{-5}$	$3.75\times10^{-4} \pm 1.24\times10^{-5}$	<0.01	↓	$4.52\times10^{-3} \pm 1.04\times10^{-4}$	$1.23\times10^{-3} \pm 5.53\times10^{-5}$	<0.01	↓	$2.31\times10^{-3} \pm 1.29\times10^{-4}$	$1.32\times10^{-3} \pm 8.65\times10^{-5}$	<0.01	↓
Amino acids, peptides, and analogues												
4-Aminobutyrate	$4.24\times10^{-4} \pm 4.11\times10^{-5}$	$1.39\times10^{-3} \pm 1.93\times10^{-5}$	<0.01	↑	$1.43\times10^{-3} \pm 2.57\times10^{-5}$	$1.95\times10^{-3} \pm 7.75\times10^{-5}$	<0.01	↑	$1.19\times10^{-3} \pm 6.13\times10^{-5}$	$1.69\times10^{-3} \pm 1.27\times10^{-4}$	<0.01	↑
5-Aminolevulinate	$1.88\times10^{-4} \pm 1.41\times10^{-5}$	$6.71\times10^{-5} \pm 3.47\times10^{-6}$	<0.01	↓	$4.16\times10^{-5} \pm 7.44\times10^{-6}$	$7.56\times10^{-5} \pm 9.66\times10^{-6}$	<0.01	↑	$1.10\times10^{-4} \pm 5.83\times10^{-6}$	$9.56\times10^{-5} \pm 9.85\times10^{-6}$	0.36	=
Alanine	$4.18\times10^{-4} \pm 2.09\times10^{-5}$	$1.96\times10^{-3} \pm 2.33\times10^{-5}$	<0.01	↑	$1.04\times10^{-3} \pm 9.56\times10^{-6}$	$1.43\times10^{-3} \pm 9.31\times10^{-5}$	<0.01	↑	$8.94\times10^{-4} \pm 6.04\times10^{-5}$	$1.04\times10^{-3} \pm 7.36\times10^{-5}$	<0.01	↑
Arginine	$2.80\times10^{-2} \pm 1.27\times10^{-3}$	$1.97\times10^{-3} \pm 2.05\times10^{-4}$	<0.01	↓	$2.59\times10^{-2} \pm 9.24\times10^{-4}$	$3.29\times10^{-2} \pm 2.23\times10^{-3}$	0.12	=	$3.17\times10^{-2} \pm 2.27\times10^{-3}$	$2.48\times10^{-2} \pm 1.42\times10^{-3}$	<0.01	↓
Asparagine	$6.69\times10^{-5} \pm 2.35\times10^{-5}$	$1.98\times10^{-4} \pm 1.67\times10^{-5}$	<0.01	↑	$1.60\times10^{-4} \pm 5.70\times10^{-6}$	$2.52\times10^{-4} \pm 2.54\times10^{-5}$	<0.01	↑	$1.97\times10^{-4} \pm 1.14\times10^{-5}$	$1.95\times10^{-4} \pm 6.07\times10^{-6}$	0.02	↓
Aspartate	$1.13\times10^{-4} \pm 2.92\times10^{-5}$	$5.38\times10^{-4} \pm 5.57\times10^{-6}$	<0.01	↑	$1.64\times10^{-4} \pm 1.59\times10^{-5}$	$1.77\times10^{-4} \pm 2.39\times10^{-5}$	0.06	=	$1.76\times10^{-4} \pm 1.22\times10^{-5}$	$2.35\times10^{-4} \pm 1.80\times10^{-5}$	<0.01	↑
Carnosine	$1.16\times10^{-4} \pm 1.21\times10^{-5}$	$9.53\times10^{-5} \pm 3.77\times10^{-6}$	<0.01	↓	$9.83\times10^{-5} \pm 2.13\times10^{-5}$	$5.42\times10^{-5} \pm 1.07\times10^{-5}$	<0.01	↓	$7.25\times10^{-5} \pm 1.01\times10^{-5}$	$9.75\times10^{-5} \pm 3.83\times10^{-6}$	<0.01	↑
Isoleucine	$3.86\times10^{-5} \pm 5.65\times10^{-6}$	$1.28\times10^{-4} \pm 6.48\times10^{-6}$	<0.01	↑	$8.88\times10^{-5} \pm 5.62\times10^{-6}$	$7.45\times10^{-5} \pm 6.19\times10^{-6}$	<0.01	↓	$6.83\times10^{-5} \pm 7.25\times10^{-6}$	$1.03\times10^{-4} \pm 9.79\times10^{-6}$	<0.01	↑
Leucine	$2.58\times10^{-4} \pm 2.24\times10^{-5}$	$6.89\times10^{-4} \pm 5.23\times10^{-6}$	<0.01	↑	$2.84\times10^{-4} \pm 2.12\times10^{-5}$	$2.96\times10^{-4} \pm 3.68\times10^{-5}$	0.02	↑	$3.42\times10^{-4} \pm 2.01\times10^{-5}$	$5.49\times10^{-4} \pm 5.04\times10^{-5}$	<0.01	↑
Methionine	$6.95\times10^{-5} \pm 4.79\times10^{-6}$	$2.22\times10^{-4} \pm 2.50\times10^{-6}$	<0.01	↑	$8.52\times10^{-5} \pm 5.19\times10^{-6}$	$9.31\times10^{-5} \pm 5.70\times10^{-6}$	0.05	↑	$9.88\times10^{-5} \pm 7.84\times10^{-6}$	$1.23\times10^{-4} \pm 7.70\times10^{-6}$	<0.01	↑
N,N-Dimethylglycine	$4.21\times10^{-5} \pm 1.76\times10^{-6}$	$4.53\times10^{-5} \pm 1.21\times10^{-6}$	0.01	↑	$1.97\times10^{-5} \pm 8.92\times10^{-7}$	$1.81\times10^{-5} \pm 1.99\times10^{-6}$	<0.01	↓	$1.26\times10^{-5} \pm 8.41\times10^{-7}$	$1.29\times10^{-5} \pm 7.47\times10^{-7}$	0.01	↑
Phenylalanine	$8.26\times10^{-5} \pm 1.45\times10^{-5}$	$4.10\times10^{-4} \pm 6.76\times10^{-6}$	<0.01	↑	$1.58\times10^{-4} \pm 7.19\times10^{-6}$	$1.44\times10^{-4} \pm 8.66\times10^{-6}$	<0.01	↓	$1.24\times10^{-4} \pm 1.87\times10^{-5}$	$2.35\times10^{-4} \pm 1.63\times10^{-5}$	<0.01	↑
Proline	$8.09\times10^{-5} \pm 6.52\times10^{-6}$	$2.16\times10^{-5} \pm 8.56\times10^{-6}$	<0.01	↓	$5.79\times10^{-4} \pm 3.04\times10^{-5}$	$1.16\times10^{-3} \pm 4.45\times10^{-5}$	<0.01	↑	$3.00\times10^{-4} \pm 2.42\times10^{-5}$	$6.23\times10^{-6} \pm 2.72\times10^{-6}$	<0.01	↓
Pyroglutamate	$1.20\times10^{-4} \pm 2.17\times10^{-5}$	$3.90\times10^{-4} \pm 9.76\times10^{-5}$	<0.01	↑	$5.31\times10^{-4} \pm 1.71\times10^{-5}$	$5.87\times10^{-4} \pm 5.55\times10^{-5}$	0.10	=	$2.43\times10^{-3} \pm 1.39\times10^{-4}$	$2.73\times10^{-3} \pm 2.12\times10^{-4}$	<0.01	↑
Sarcosine	$1.20\times10^{-5} \pm 2.44\times10^{-6}$	$9.15\times10^{-6} \pm 2.62\times10^{-6}$	0.12	=	$3.20\times10^{-5} \pm 8.24\times10^{-7}$	$2.07\times10^{-5} \pm 3.84\times10^{-6}$	<0.01	↓	$1.78\times10^{-5} \pm 1.65\times10^{-6}$	$3.18\times10^{-5} \pm 2.64\times10^{-6}$	<0.01	↑

Tryptophan	$5.26\times10^{-5}\pm2.16\times10^{-5}$	$1.57\times10^{-4}\pm1.33\times10^{-5}$	<0.01	↑	$6.78\times10^{-5}\pm3.10\times10^{-6}$	$6.55\times10^{-5}\pm5.67\times10^{-6}$	<0.01	↓	$5.12\times10^{-5}\pm1.34\times10^{-5}$	$7.21\times10^{-5}\pm1.05\times10^{-5}$	0.02	↑
Tyrosine	$4.40\times10^{-5}\pm6.97\times10^{-6}$	$4.46\times10^{-4}\pm4.98\times10^{-6}$	<0.01	↑	$1.27\times10^{-4}\pm8.64\times10^{-6}$	$1.29\times10^{-4}\pm9.17\times10^{-6}$	0.01	↑	$8.45\times10^{-5}\pm9.40\times10^{-6}$	$1.34\times10^{-4}\pm5.94\times10^{-6}$	<0.01	↑
Ornithine	$7.18\times10^{-4}\pm1.46\times10^{-4}$	$5.57\times10^{-4}\pm7.69\times10^{-5}$	0.07	=	$3.09\times10^{-3}\pm2.69\times10^{-4}$	$2.67\times10^{-4}\pm7.71\times10^{-5}$	<0.01	↓	$2.14\times10^{-4}\pm6.60\times10^{-5}$	$2.75\times10^{-5}\pm1.59\times10^{-5}$	<0.01	↓
Valine	$4.47\times10^{-5}\pm1.18\times10^{-5}$	$2.51\times10^{-4}\pm5.27\times10^{-6}$	<0.01	↑	$1.41\times10^{-4}\pm7.09\times10^{-6}$	$1.37\times10^{-4}\pm1.55\times10^{-5}$	<0.01	↓	$1.13\times10^{-4}\pm1.16\times10^{-5}$	$1.45\times10^{-4}\pm1.29\times10^{-5}$	<0.01	↑
Carbohydrates and carbohydrate conjugates												
Arabinose	$6.93\times10^{-3}\pm7.90\times10^{-4}$	$1.49\times10^{-3}\pm3.60\times10^{-4}$	<0.01	↓	$6.91\times10^{-3}\pm6.48\times10^{-4}$	$8.89\times10^{-3}\pm7.75\times10^{-4}$	0.22	=	$7.28\times10^{-3}\pm5.00\times10^{-4}$	$6.99\times10^{-3}\pm1.57\times10^{-4}$	0.05	=
Fructose	$2.95\times10^{-3}\pm1.82\times10^{-4}$	$3.27\times10^{-3}\pm1.18\times10^{-4}$	0.01	↑	$1.57\times10^{-3}\pm5.14\times10^{-5}$	$2.67\times10^{-3}\pm1.99\times10^{-4}$	<0.01	↑	$2.19\times10^{-3}\pm1.19\times10^{-4}$	$2.33\times10^{-3}\pm2.05\times10^{-4}$	<0.01	↑
Fucose	$6.34\times10^{-3}\pm2.85\times10^{-4}$	$1.05\times10^{-2}\pm5.83\times10^{-4}$	<0.01	↑	$3.08\times10^{-3}\pm2.90\times10^{-4}$	$5.75\times10^{-3}\pm3.46\times10^{-4}$	<0.01	↑	$5.01\times10^{-3}\pm1.65\times10^{-4}$	$4.60\times10^{-3}\pm2.55\times10^{-4}$	0.18	=
Galactose	$5.79\times10^{-3}\pm1.82\times10^{-4}$	$6.26\times10^{-3}\pm3.93\times10^{-4}$	0.05	=	$4.16\times10^{-3}\pm3.65\times10^{-4}$	$2.41\times10^{-4}\pm7.12\times10^{-5}$	<0.01	↓	$3.32\times10^{-3}\pm2.99\times10^{-4}$	$3.06\times10^{-3}\pm1.76\times10^{-4}$	0.63	=
Glucose	$4.63\times10^{-3}\pm2.97\times10^{-4}$	$4.73\times10^{-3}\pm7.54\times10^{-4}$	0.76	=	$2.68\times10^{-3}\pm6.56\times10^{-5}$	$4.30\times10^{-3}\pm3.73\times10^{-4}$	<0.01	↑	$3.52\times10^{-3}\pm2.10\times10^{-4}$	$3.07\times10^{-3}\pm2.20\times10^{-4}$	<0.01	↓
Xylose	$1.32\times10^{-3}\pm9.61\times10^{-5}$	$1.65\times10^{-3}\pm1.25\times10^{-4}$	<0.01	↑	$7.19\times10^{-4}\pm2.88\times10^{-5}$	$1.29\times10^{-3}\pm7.53\times10^{-5}$	<0.01	↑	$1.11\times10^{-3}\pm6.89\times10^{-5}$	$9.78\times10^{-4}\pm4.72\times10^{-5}$	0.30	=
Organic acids and derivatives												
2-Hydroxybutyrate	$4.45\times10^{-4}\pm2.95\times10^{-5}$	$3.01\times10^{-4}\pm1.58\times10^{-6}$	<0.01	↓	$6.96\times10^{-4}\pm4.04\times10^{-5}$	$9.00\times10^{-4}\pm5.68\times10^{-5}$	0.12	=	$1.08\times10^{-3}\pm4.17\times10^{-5}$	$7.65\times10^{-4}\pm5.42\times10^{-5}$	<0.01	↓
2-Hydroxyglutarate	$1.84\times10^{-3}\pm1.04\times10^{-4}$	$5.91\times10^{-4}\pm2.93\times10^{-5}$	<0.01	↓	$1.92\times10^{-3}\pm5.64\times10^{-5}$	$1.70\times10^{-3}\pm7.07\times10^{-5}$	<0.01	↓	$3.02\times10^{-3}\pm2.33\times10^{-4}$	$1.31\times10^{-3}\pm1.15\times10^{-4}$	<0.01	↓
trans-Aconitate	$5.02\times10^{-5}\pm6.85\times10^{-6}$	$7.44\times10^{-5}\pm7.90\times10^{-6}$	<0.01	↑	$6.17\times10^{-5}\pm6.44\times10^{-6}$	$7.75\times10^{-5}\pm6.27\times10^{-6}$	0.53	=	$4.82\times10^{-5}\pm1.32\times10^{-5}$	$6.04\times10^{-5}\pm6.93\times10^{-6}$	<0.01	↑
2-Hydroxyisovalerate	$5.29\times10^{-5}\pm8.91\times10^{-6}$	$6.28\times10^{-6}\pm2.81\times10^{-6}$	<0.01	↓	$5.86\times10^{-5}\pm7.28\times10^{-6}$	$5.93\times10^{-5}\pm1.34\times10^{-5}$	0.16	=	$1.06\times10^{-4}\pm4.68\times10^{-6}$	$7.11\times10^{-5}\pm7.01\times10^{-6}$	<0.01	↓
2-Oxoglutarate	$7.92\times10^{-4}\pm5.69\times10^{-5}$	$2.02\times10^{-4}\pm2.68\times10^{-5}$	<0.01	↓	$2.55\times10^{-4}\pm1.29\times10^{-5}$	$6.44\times10^{-4}\pm3.89\times10^{-5}$	<0.01	↑	$8.19\times10^{-4}\pm5.95\times10^{-5}$	$4.92\times10^{-4}\pm3.13\times10^{-5}$	<0.01	↓
3-Hydroxybutyrate	$2.35\times10^{-4}\pm1.69\times10^{-5}$	$1.20\times10^{-4}\pm1.10\times10^{-5}$	<0.01	↓	$3.78\times10^{-4}\pm2.03\times10^{-5}$	$5.15\times10^{-4}\pm2.70\times10^{-5}$	<0.01	↑	$5.41\times10^{-4}\pm3.02\times10^{-5}$	$4.16\times10^{-4}\pm2.95\times10^{-5}$	<0.01	↑
Acetate	$4.48\times10^{-3}\pm2.38\times10^{-4}$	$3.07\times10^{-3}\pm1.83\times10^{-5}$	<0.01	↓	$3.38\times10^{-3}\pm5.21\times10^{-5}$	$4.77\times10^{-3}\pm2.46\times10^{-4}$	<0.01	↑	$2.81\times10^{-3}\pm1.93\times10^{-4}$	$1.62\times10^{-3}\pm1.14\times10^{-4}$	<0.01	↓
Butyrate	$2.91\times10^{-4}\pm2.13\times10^{-5}$	$7.62\times10^{-5}\pm1.81\times10^{-6}$	<0.01	↓	$1.33\times10^{-4}\pm8.19\times10^{-6}$	$2.88\times10^{-4}\pm2.41\times10^{-5}$	<0.01	↑	$1.60\times10^{-4}\pm1.13\times10^{-5}$	$8.30\times10^{-5}\pm5.85\times10^{-6}$	<0.01	↓
Formate	$1.01\times10^{-4}\pm1.20\times10^{-5}$	$8.16\times10^{-5}\pm3.48\times10^{-6}$	<0.01	↓	$1.14\times10^{-4}\pm9.29\times10^{-6}$	$7.51\times10^{-5}\pm2.77\times10^{-5}$	0.01	↓	$7.44\times10^{-5}\pm1.14\times10^{-5}$	$4.10\times10^{-5}\pm1.26\times10^{-5}$	<0.01	↓
Fumarate	$3.92\times10^{-5}\pm5.47\times10^{-6}$	$4.03\times10^{-4}\pm5.56\times10^{-6}$	<0.01	↑	$3.83\times10^{-5}\pm5.97\times10^{-6}$	$5.19\times10^{-5}\pm3.35\times10^{-6}$	0.15	=	$4.77\times10^{-5}\pm4.74\times10^{-6}$	$7.33\times10^{-5}\pm8.67\times10^{-6}$	<0.01	↑
Galactarate	$1.12\times10^{-4}\pm7.06\times10^{-6}$	$4.00\times10^{-4}\pm1.30\times10^{-5}$	<0.01	↑	$4.81\times10^{-5}\pm3.60\times10^{-6}$	$1.09\times10^{-4}\pm6.68\times10^{-6}$	<0.01	↑	$1.49\times10^{-4}\pm9.70\times10^{-6}$	$3.86\times10^{-5}\pm6.30\times10^{-6}$	<0.01	↓
Galactonate	$5.13\times10^{-4}\pm7.87\times10^{-5}$	$9.49\times10^{-5}\pm2.99\times10^{-5}$	<0.01	↓	$3.33\times10^{-4}\pm2.71\times10^{-5}$	$1.16\times10^{-3}\pm1.14\times10^{-4}$	<0.01	↑	$7.94\times10^{-4}\pm5.10\times10^{-5}$	$1.76\times10^{-4}\pm1.01\times10^{-5}$	<0.01	↓

Isobutyrate	$1.31\times10^{-3}\pm7.02\times10^{-5}$	$4.63\times10^{-4}\pm5.73\times10^{-6}$	<0.01	↓	$1.05\times10^{-3}\pm3.08\times10^{-5}$	$1.36\times10^{-3}\pm1.02\times10^{-4}$	0.03	↑	$1.22\times10^{-3}\pm8.95\times10^{-5}$	$1.05\times10^{-3}\pm7.27\times10^{-5}$	0.075	=
Isocaproate	$1.49\times10^{-4}\pm6.91\times10^{-6}$	$3.24\times10^{-5}\pm1.79\times10^{-6}$	<0.01	↓	$5.62\times10^{-5}\pm4.49\times10^{-6}$	$1.34\times10^{-4}\pm1.31\times10^{-5}$	<0.01	↑	$7.16\times10^{-5}\pm7.43\times10^{-6}$	$3.00\times10^{-5}\pm3.15\times10^{-7}$	<0.01	↓
Isovalerate	$1.28\times10^{-3}\pm6.71\times10^{-5}$	$4.95\times10^{-4}\pm2.59\times10^{-6}$	<0.01	↓	$6.17\times10^{-4}\pm1.80\times10^{-5}$	$8.69\times10^{-4}\pm6.09\times10^{-5}$	<0.01	↑	$8.23\times10^{-4}\pm4.37\times10^{-5}$	$5.46\times10^{-4}\pm4.11\times10^{-5}$	<0.01	↓
Lactate	$2.26\times10^{-3}\pm1.17\times10^{-4}$	$3.95\times10^{-3}\pm2.38\times10^{-4}$	<0.01	↑	$2.18\times10^{-2}\pm1.56\times10^{-3}$	$1.70\times10^{-3}\pm1.77\times10^{-4}$	<0.01	↓	$1.39\times10^{-3}\pm1.18\times10^{-4}$	$1.27\times10^{-3}\pm9.28\times10^{-5}$	0.32	=
Levulinate	$2.77\times10^{-5}\pm1.80\times10^{-6}$	$9.28\times10^{-6}\pm1.46\times10^{-6}$	<0.01	↓	$2.71\times10^{-5}\pm1.05\times10^{-6}$	$6.85\times10^{-6}\pm8.91\times10^{-7}$	<0.01	↓	$3.58\times10^{-5}\pm2.60\times10^{-6}$	$6.43\times10^{-6}\pm1.65\times10^{-6}$	<0.01	↓
Malate	$3.77\times10^{-2}\pm9.64\times10^{-3}$	$3.82\times10^{-2}\pm2.63\times10^{-4}$	0.42	=	$6.06\times10^{-4}\pm2.05\times10^{-4}$	$2.18\times10^{-2}\pm1.79\times10^{-3}$	<0.01	↑	$2.97\times10^{-2}\pm1.84\times10^{-3}$	$2.41\times10^{-2}\pm2.06\times10^{-3}$	<0.01	↓
Malonate	$2.93\times10^{-5}\pm3.00\times10^{-6}$	$5.18\times10^{-5}\pm5.25\times10^{-6}$	<0.01	↑	$6.43\times10^{-5}\pm3.15\times10^{-6}$	$5.84\times10^{-5}\pm4.89\times10^{-6}$	<0.01	↓	$6.45\times10^{-5}\pm3.79\times10^{-6}$	$6.85\times10^{-5}\pm4.76\times10^{-6}$	<0.01	↑
Pyruvate	$7.72\times10^{-4}\pm1.15\times10^{-4}$	$8.81\times10^{-4}\pm4.93\times10^{-5}$	0.10	=	$2.95\times10^{-4}\pm1.24\times10^{-5}$	$1.59\times10^{-3}\pm1.75\times10^{-4}$	<0.01	↑	$1.26\times10^{-3}\pm1.82\times10^{-4}$	$1.82\times10^{-3}\pm3.21\times10^{-4}$	<0.01	↑
Succinate	$7.17\times10^{-3}\pm4.06\times10^{-4}$	$2.92\times10^{-3}\pm1.96\times10^{-5}$	<0.01	↓	$2.69\times10^{-3}\pm3.44\times10^{-5}$	$5.98\times10^{-3}\pm3.18\times10^{-4}$	<0.01	↑	$1.01\times10^{-2}\pm6.98\times10^{-4}$	$5.02\times10^{-3}\pm3.83\times10^{-4}$	<0.01	↓
Tartrate	$7.73\times10^{-6}\pm1.89\times10^{-6}$	$3.91\times10^{-6}\pm4.84\times10^{-7}$	<0.01	↓	$9.08\times10^{-6}\pm1.53\times10^{-6}$	$4.33\times10^{-5}\pm6.21\times10^{-6}$	<0.01	↑	$1.67\times10^{-5}\pm1.59\times10^{-6}$	$6.17\times10^{-6}\pm4.86\times10^{-7}$	<0.01	↓
Others												
1,3-Dihydroxyacetone	$3.85\times10^{-5}\pm5.52\times10^{-6}$	$1.81\times10^{-5}\pm2.40\times10^{-6}$	<0.01	↓	$5.22\times10^{-5}\pm6.49\times10^{-6}$	$7.92\times10^{-5}\pm4.29\times10^{-6}$	0.01	↑	$5.00\times10^{-5}\pm5.35\times10^{-6}$	$3.79\times10^{-5}\pm3.56\times10^{-6}$	0.04	↓
Acetoin	$1.63\times10^{-4}\pm9.18\times10^{-6}$	$8.63\times10^{-5}\pm6.64\times10^{-6}$	<0.01	↓	$8.74\times10^{-5}\pm1.03\times10^{-5}$	$4.58\times10^{-5}\pm6.11\times10^{-6}$	<0.01	↓	$2.65\times10^{-4}\pm1.87\times10^{-5}$	$9.12\times10^{-5}\pm8.76\times10^{-6}$	<0.01	↓
Acetone	$4.28\times10^{-4}\pm3.83\times10^{-5}$	$8.31\times10^{-5}\pm2.87\times10^{-6}$	<0.01	↓	$2.87\times10^{-4}\pm1.58\times10^{-5}$	$9.05\times10^{-5}\pm7.00\times10^{-6}$	<0.01	↓	$4.33\times10^{-4}\pm3.54\times10^{-5}$	$8.97\times10^{-5}\pm9.50\times10^{-6}$	<0.01	↓
Ascorbate	$1.11\times10^{-3}\pm1.61\times10^{-4}$	$3.68\times10^{-4}\pm1.05\times10^{-4}$	<0.01	↓	$3.18\times10^{-3}\pm8.49\times10^{-5}$	$4.22\times10^{-3}\pm3.62\times10^{-4}$	0.02	↑	$2.41\times10^{-3}\pm1.45\times10^{-4}$	$1.90\times10^{-3}\pm1.41\times10^{-4}$	<0.01	↓
Choline	$3.28\times10^{-4}\pm2.01\times10^{-5}$	$5.40\times10^{-4}\pm3.65\times10^{-6}$	<0.01	↑	$5.49\times10^{-4}\pm1.46\times10^{-5}$	$6.09\times10^{-4}\pm3.86\times10^{-5}$	0.03	↑	$4.09\times10^{-4}\pm2.84\times10^{-5}$	$4.12\times10^{-4}\pm2.98\times10^{-5}$	<0.01	↑
Cytidine	$1.42\times10^{-4}\pm9.46\times10^{-6}$	$1.49\times10^{-4}\pm1.47\times10^{-5}$	0.41	=	$1.17\times10^{-4}\pm3.21\times10^{-6}$	$1.14\times10^{-4}\pm1.72\times10^{-5}$	<0.01	↓	$1.54\times10^{-4}\pm8.34\times10^{-6}$	$2.11\times10^{-4}\pm1.64\times10^{-5}$	<0.01	↑
Dimethylamine	$4.51\times10^{-6}\pm6.79\times10^{-7}$	$3.06\times10^{-6}\pm1.06\times10^{-6}$	0.04	↓	$1.17\times10^{-5}\pm5.98\times10^{-7}$	$8.35\times10^{-6}\pm8.10\times10^{-7}$	<0.01	↓	$6.45\times10^{-6}\pm7.68\times10^{-7}$	$9.82\times10^{-6}\pm9.06\times10^{-7}$	<0.01	↑
Ethanolamine	$2.19\times10^{-4}\pm1.39\times10^{-5}$	$2.70\times10^{-4}\pm1.66\times10^{-5}$	<0.01	↑	$3.73\times10^{-4}\pm1.48\times10^{-5}$	$3.53\times10^{-4}\pm1.47\times10^{-5}$	<0.01	↓	$2.42\times10^{-4}\pm1.45\times10^{-5}$	$2.41\times10^{-4}\pm2.21\times10^{-5}$	0.04	↓
Gallate	$2.47\times10^{-5}\pm3.80\times10^{-6}$	$1.97\times10^{-5}\pm1.93\times10^{-6}$	0.04	↓	$2.54\times10^{-5}\pm3.67\times10^{-6}$	$1.78\times10^{-5}\pm2.73\times10^{-6}$	<0.01	↓	$6.64\times10^{-5}\pm3.95\times10^{-6}$	$3.04\times10^{-5}\pm4.50\times10^{-6}$	<0.01	↓
Hydroxyacetone	$2.68\times10^{-5}\pm3.71\times10^{-6}$	$2.14\times10^{-5}\pm5.89\times10^{-6}$	0.12	=	$5.27\times10^{-6}\pm2.29\times10^{-6}$	$1.71\times10^{-4}\pm2.61\times10^{-5}$	<0.01	↑	$6.68\times10^{-5}\pm3.95\times10^{-6}$	$1.90\times10^{-5}\pm2.12\times10^{-6}$	<0.01	↓
N-Nitrosodimethylamine	$1.35\times10^{-5}\pm2.73\times10^{-6}$	$2.12\times10^{-5}\pm2.14\times10^{-6}$	<0.01	↑	$1.41\times10^{-5}\pm1.69\times10^{-6}$	$1.72\times10^{-5}\pm2.78\times10^{-6}$	0.87	=	$9.82\times10^{-6}\pm1.94\times10^{-6}$	$1.49\times10^{-5}\pm1.14\times10^{-6}$	<0.01	↑
Oxypurinol	$8.91\times10^{-5}\pm1.25\times10^{-5}$	$1.87\times10^{-4}\pm1.56\times10^{-5}$	<0.01	↑	$1.17\times10^{-4}\pm6.21\times10^{-6}$	$1.18\times10^{-4}\pm1.47\times10^{-5}$	<0.01	↑	$1.13\times10^{-4}\pm1.81\times10^{-5}$	$2.00\times10^{-4}\pm8.27\times10^{-6}$	<0.01	↑
Uridine	$8.87\times10^{-5}\pm1.32\times10^{-5}$	$1.78\times10^{-4}\pm1.17\times10^{-5}$	<0.01	↑	$1.68\times10^{-4}\pm5.22\times10^{-6}$	$1.47\times10^{-4}\pm1.84\times10^{-5}$	<0.01	↓	$1.36\times10^{-4}\pm1.42\times10^{-5}$	$1.96\times10^{-4}\pm1.20\times10^{-5}$	<0.01	↑
Xanthine	$3.80\times10^{-5}\pm6.56\times10^{-6}$	$1.13\times10^{-4}\pm1.00\times10^{-5}$	<0.01	↑	$6.91\times10^{-5}\pm3.05\times10^{-6}$	$6.17\times10^{-5}\pm4.38\times10^{-6}$	<0.01	↓	$5.81\times10^{-5}\pm4.00\times10^{-6}$	$1.12\times10^{-4}\pm8.22\times10^{-6}$	<0.01	↑

Hypoxanthine	$3.01\times10^{-5}\pm8.31\times10^{-6}$	$1.02\times10^{-4}\pm3.76\times10^{-5}$	0.01	↑	$2.15\times10^{-5}\pm5.28\times10^{-6}$	$2.21\times10^{-5}\pm5.27\times10^{-6}$	0.30	=	$4.29\times10^{-5}\pm5.93\times10^{-6}$	$3.38\times10^{-5}\pm1.19\times10^{-5}$	0.57	=

Data are displayed as mean ± standard deviation (n = 5).

* "↑" represents significant increase, "↓" represents significant decrease, "=" represents no significant difference ($p < 0.05$).
